# Analysis of microRNA reveals cleistogamous and chasmogamous floret divergence in dimorphic plant

**DOI:** 10.1038/s41598-018-24477-x

**Published:** 2018-04-19

**Authors:** Fan Wu, Daiyu Zhang, Blaise Pascal Muvunyi, Qi Yan, Yufei Zhang, Zhuanzhuan Yan, Mingshu Cao, Yanrong Wang, Jiyu Zhang

**Affiliations:** 1 State Key Laboratory of Grassland Agro-ecosystems, Key Laboratory of Grassland Livestock Industry Innovation, Ministry of Agriculture, College of Pastoral Agriculture Science and Technology, Lanzhou University, Lanzhou 730020, China; 2 AgResearch Limited, Grassland Research Centre, Palmerston North 4442, New Zealand

## Abstract

*Cleistogenes songorica*, a grass species that exhibits two spatially different type of inflorescence, chastogamy (CH), flowers localized at the top, and cleistogamy (CL) flowers embedded in leaf sheath. This study aimed at dissecting reasons underlying these distinct floral development patterns at morphological and microRNA level. Phenotyping for CH and CL was conducted and four small RNA libraries were constructed from the CH and CL flowers for high-throughput sequencing to identify the differentiated miRNAs. As results, spikelet, stigma, anther, lemma and lodicule length of CH flowers were found larger than that of CL, and so was seed setting. Also, 17 flower-related differential expression miRNAs were identified which were associated with floral organ development and morphogenesis, and the flower development. Further results showed that miR159a.1-CL3996.Contig2 pair was related to anther development, miR156a-5p-CL1954.Contig2 was linked to response to high light intensity, miR408-3p/miR408d-Unigene429 was related to pollination and Unigene429 positively regulated flower development. To our knowledge, this is the first study on differential miRNA accumulation between CH and CL flowers and our study serves as a foundation to the future elucidation of regulatory mechanisms of miRNAs in the divergent development of CL and CH flowers in a single plant.

## Introduction

Chasmogamy (CH), an open-pollinated reproduction mechanism, is typical in grasses, with open flowers where the palea and the lemma are compelled separation thus exposing the pistil to disperse anemophilous pollen^1^. Cleistogamy (CL) flowers keep closed until they have been fertilized^2^ and this type of flower reproductive system has been found in about 700 plant species^3–5^. At present, cleistogamy was recognized in three forms including complete self-pollination, mutagenic cleistogamy and both closed and opened pollination (dimorphic)^4^. Cleistogamous plants, with strong ability to adapt to adversity^6^, benefit from guaranteeing mating success and genetic uniformity while chasmogamy is beneficial for plants to maintain genetic variation resource^7^. CL flowers usually develop earlier but end up with smaller flowers, and compared with CH flowers, CL flowers were considered as the improved form in dimorphic plants^8,9^. Barley is a dominated self-pollination species. Nonetheless, CH still exists in some wild barley species. In some natural barley population, the paleas and the lemmas are closed during the anthesis which is of the CL type. Furthermore, the development of CH or CL flowers were influenced by environment factors, such as drought stress, salt stress, nutritional imbalance, photoperiod, etc^3,10^. *Viola philippica* develops CH flowers under short daylight, however, the intermediate CL and CH flowers are induced with prolonging photoperiod, and the size and number of petals and stamens are smaller than that of the naturally developed CH flowers^11^.

*C*. *songorica*, belongs to the Poaceae family, produces both CH and CL flowers on the same individual plants that appear in different positions (Fig. S1). *C*. *songorica* is an important forage grass and ecological grass and grows in saline, semi-arid and desert areas of Northwest China, such as Inner Mongolia where average annual rainfall is 110 mm^12^. To study the drought mechanism of *C*. *songorica*, leaf and root Expression Sequence Tag (EST) resources have been used to investigate drought stress-responsive genes^13^, and some genes were transformed into *Arabidopsis thaliana* and alfalfa to confirm and enhance the stress tolerance^14–17^. However, how *C*. *songorica*-specific CL phenotype is controlled by genes, environment conditions or their interactions is still unknown.

A plant complex phenotype is usually regulated by internal genes and external environment factors. The regulation mechanism at the level of gene expression have been studied in different dimorphic CL plants^10,12^. Moreover, the CL is reported being controlled by a single recessive gene (*cly1*)^7^. MicroRNAs (miRNAs), a class of evolutionarily conserved endogenous single-stranded small RNA with 20–24 nt in length, play important roles in the regulation of phenotypes at the post-transcriptional level in many eukaryotes^18–20^. Through regulating the genes expression, miRNAs, as important regulators, control plant growing and developing, in this way to encode transcription factors and regulatory proteins^21,22^.

miRNAs have been reported to be involved in plant flower buds development^23,24^, leaf development^25^, vegetative growth of leaf, root and stem, and reproductive growth of flower^26^. The flower related gene regulatory networks and mechanisms have been investigated in some species. For example, *Petunia* has been studied and found that the flower development related miRNAs were closely associated with *Phy-BLIND* which controlled the spatial restriction of homeotic class C genes to the inner floral whorls^27,28^. Forty-four miRNAs from young flower buds were identified to be associated with 140 *MIR* loci in *Petunia axillaris*^24^. In *Arabidopsis*, miR156 regulates miR172 expression and they co-regulate the stability of juvenile and adult phases^29^. Also, in *Arabidopsis*, miR172-mediated represses the expression of TARGET OF EAT 3 (TOE3) gene, and miR156 targets SQUAMOSA PROMOTER BINDING PROTEIN-LIKE 3 directly to activate TOE3 expression. That study demonstrated the interaction between miR156 and miR172 in floral patterning^30^. A previous study indicated that CL phenotype of barley and rice was caused by lack lodicules or defect lodicules, the reason for this condition is due to the mutation of floral development related genes, like *Cleistogamy* (*Cly1*). miRNA172 causes the down-regulation of barley *Cly1*, as a result lodicules growth is restrained, and CL is induced^7^. Even though the differential gene expression patterns of natural dimorphic cleistogamy plants have been studied in *Pseudostellaria heterophylla*^10^ and *Viola philippica*^12^, the miRNAs involved in flower development of dimorphic plant remains unknown. *C*. *songorica*, as a model dimorphic cleistogamous plant, could be used to investigate both miRNAs and mRNA expression in flowering processes.

In this report, we performed Illumina Hiseq based DNA sequencing to investigate miRNAs variations in *C*. *songorica*. We constructed small RNA libraries using samples from CH and three different positions (upper, middle, bottom) from CL denoted as CL_U, CL_M and CL_B, respectively. The aims of this study include (1) to report the morphological differences of CH and CL in seeds, florets, lodicules and pollen grains; (2) to characterize miRNAs in CH and CL flowers and provide platform for further investigation of specific miRNAs in various biological processes; (3) to investigate the miRNAs and targets interaction in order to illuminate the potential miRNA-mediated regulatory mechanism on chasmogamy and cleistogamy in *C*. *songorica*. As a dimorphic species, *C*. *songorica* can adapt to severe drought environment. Therefore, the differential accumulation of miRNA between CH and CL flowers will also provide novel insights into the development of dimorphism and the molecular basis of drought resistance.

## Results

### Seed formation in different positions

**Figure 1:**
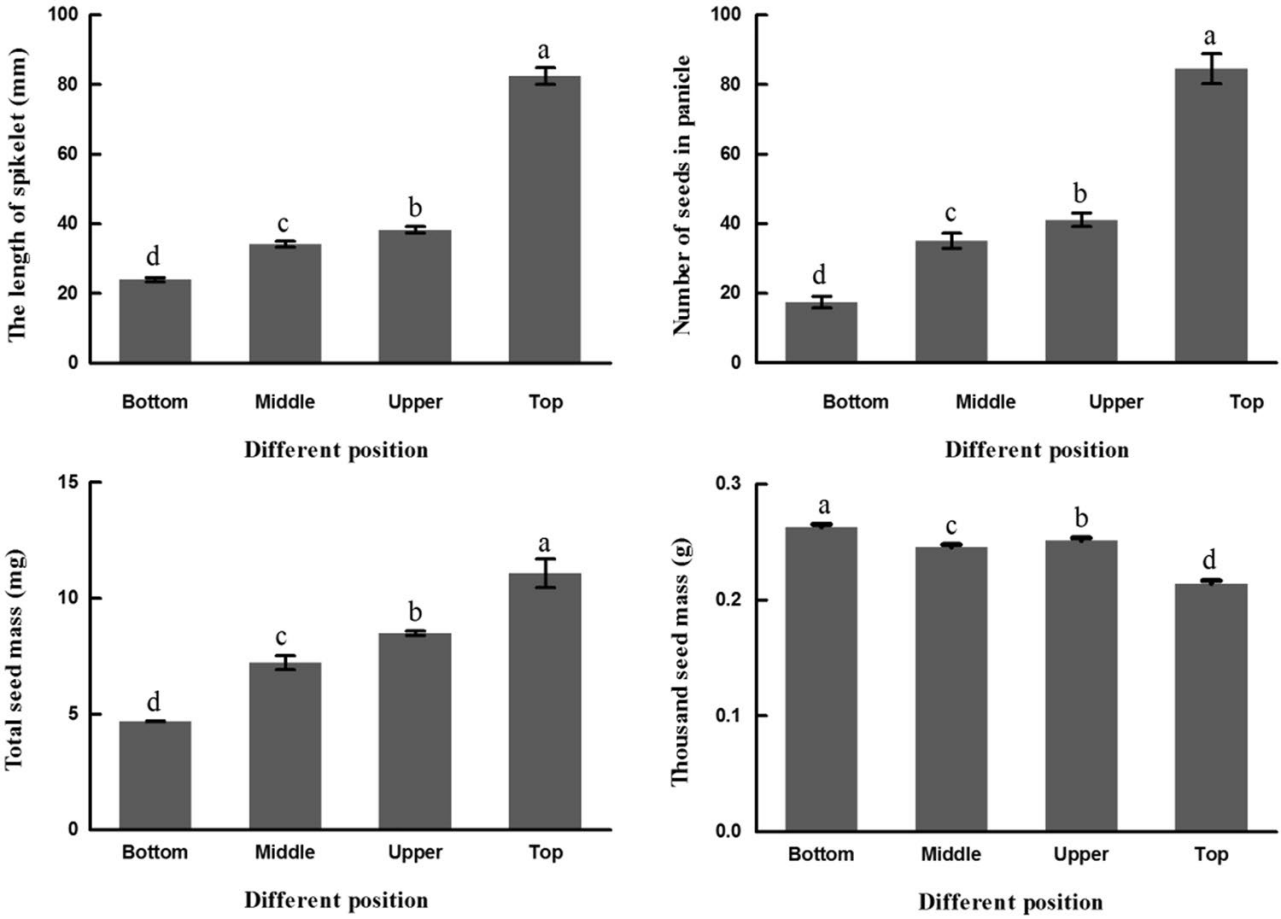
The length of different nodes spikelet, number of seeds, total seed mass and thousand seed mass along *C*. *songorica* tillers. Nodes with seeds were numbered from the bottom to the top. *P* < 0.05.

Spikelet length, seed number and seed mass were significantly divergent (*P* < 0.05) between CL and CH (Fig. 1). There was progressive increasing in seed number, seed mass and length of spikelet from the bottom to the top part of the internodes. For different position of a tiller, the bottom spikelet length was the smallest (mean value 21.46 mm), the top spikelet length was the largest (mean value 75.04) and the CH spikelet length was far larger than that of CL. The seed number showed the same trend of spikelet length. In brief, the seed number and spikelet length in the CH position were the largest, whereas the thousand-seed mass was opposite.

### The morphology of CH and CL flowers

**Figure 2:**
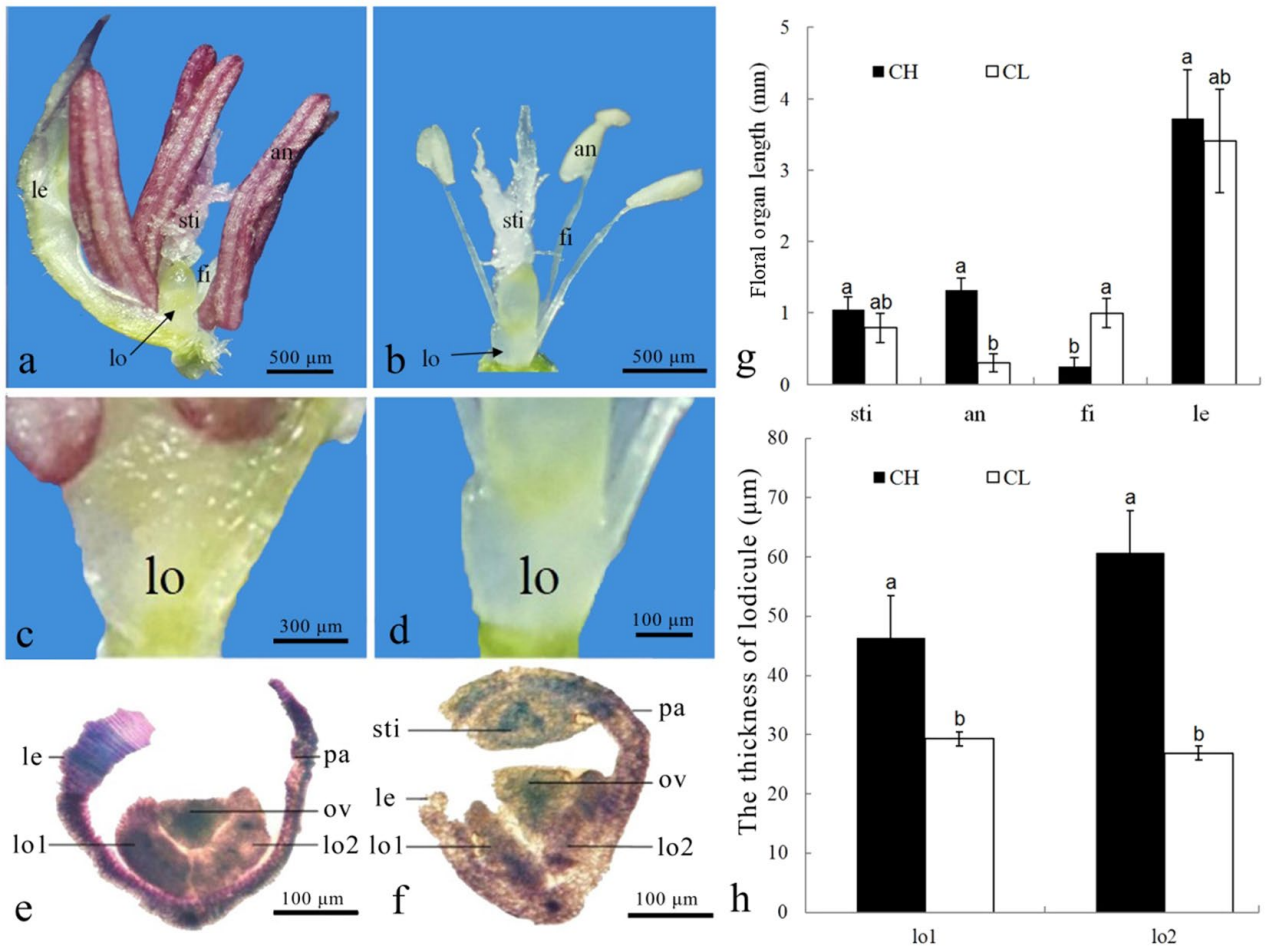
*C*. *songorica* CH and CL floral organ morphological variations. (**a**) Morphology of CH flowers. (**b**) Morphology of CL flowers. (**c**,**d**) Lodicules of CH and CL flower. (**e**,**f**) The cross sections of CL and CH lodicules. (**g**) Length of CH and CL floral organ. (**h**) The thickness of CH and CL lodicules.lo1, the first lodicule; lo2, the second lodicule; ov, overy; le, lemma; pa, palea; sti, stigma; an, anthers; fi, filaments.

*C*. *songorica* develops both CH and CL flowers, but there were some morphological difference between CH floral organs and CL floral organs (Figs 2 and S2). Five floral developmental stages were defined based on the color and the shape of CH and CL anther. Floral primordia was defined as the first stage (floret primordium stage), and floral organogenesis phase was defined as the second stage (white anther stage). Apparently, there were no obvious differences in the first and second stages of CH and CL flowers (Fig. S2). However, at the third stage (green anther stage), CH flowers formed clear green anthers with short filaments, but CL floral organ just bigger than the previous stage (Fig. S2c and h). At the fourth stage (yellow anther stage), color of CH anther became light yellow (Fig. S2d). At the fifth stage (purple anther stage), the color of CH anther became purple and the CL anther was still white, CH and CL flowers reached maximum by swelling. However, the anther of CL was obviously smaller than the previous stage, which means the pollination of CL flowers have been completed (Fig. S2e and j).

CH and CL flowers develop the same number floral organs, while organs size of CH and CL flowers was different (Fig. 2). CH and CL flowers were composed of one lemma, one palea, two lodicules, three stamens, two feathered stigmas and one ovary. The length of CH stigma, anther and lemma were larger than those of CL flowers. Conversely, the filament of CL stamens was well developed while that of CH flowers was nearly undetectable (Fig. 2a and b). Lodicule size differed markedly between CH and CL flowers (Fig. 2c–f). During maturity stage of anther, CH lodicules were at least twice times larger than that of CL (Fig. 2c–d). The most pronounced difference was lodicule depth of CH and CL (Fig. 2e–f). The thickness of the first and second lodicules in a CH flower was swelling to be largest during anthesis, at that point they were 1.58 and 2.25 times larger than CL flowers, respectively.

### Scanning electron microscopy (SEM) observation and morphometric analysis of pollen

**Figure 3:**
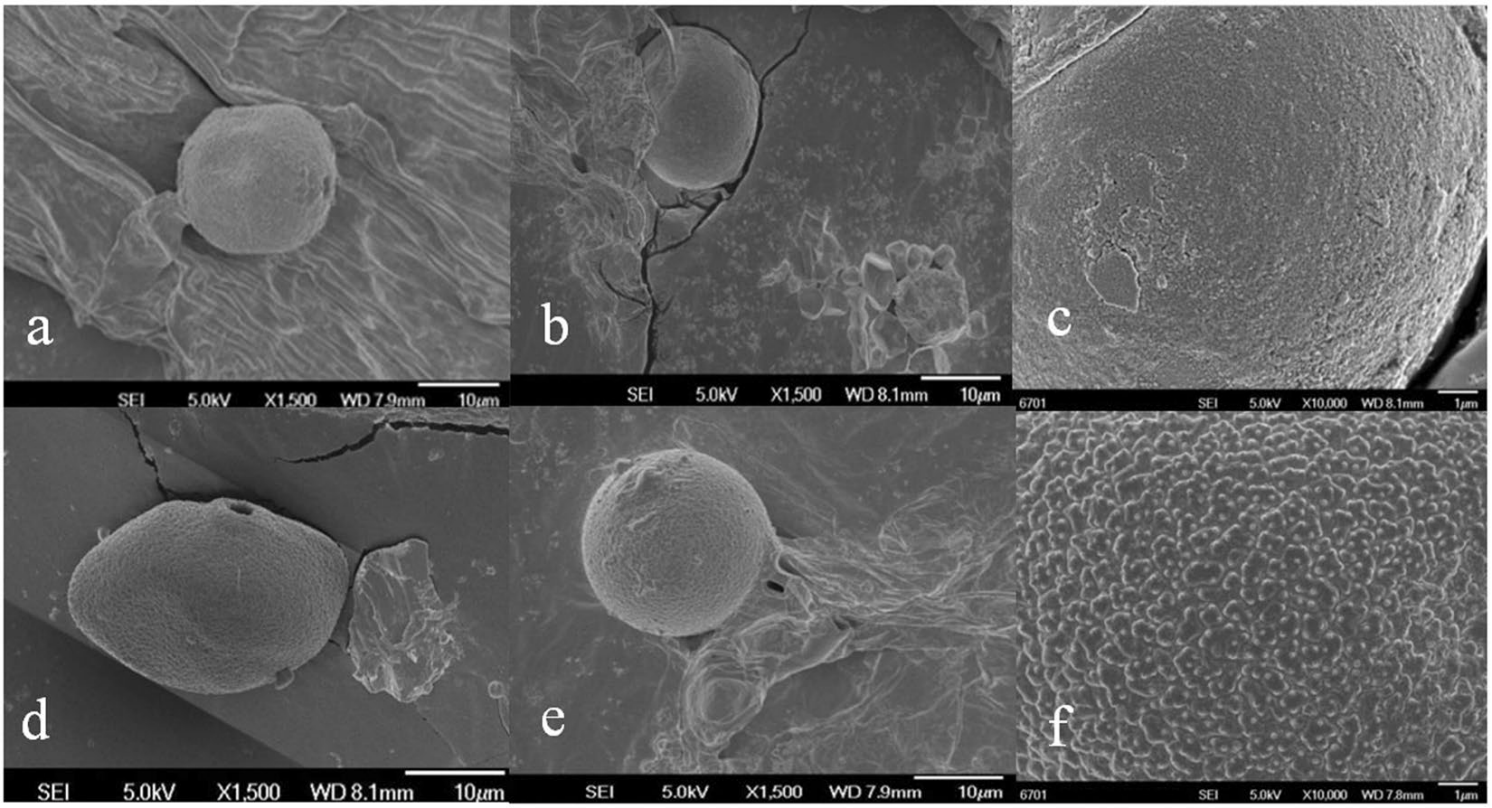
Micrographs of CH and CL pollen grain. (**a**–**d**) Shape of CH and CL pollen. (**b**,**c**) Polar view of CH and CL pollen. (**e**,**f**) Ornamentation of CH and CL pollen. The scale bar in (**a**,**b**,**d** and **e**) is 10 μm, and in (**c)** and (**f**) is1 μm.

The micro-morphology of pollen grains of CH and CL were observed using scanning electron microscope (SEM). Both CH and CL pollen grains have one germination aperture, and polar view of pollen grains were circular (Fig. 3a,b,d and e). The equatorial view of CH pollen grains was oblate spheroidal, with polar axis lengths (P) 28.26 ± 1.31 μm and equatorial diameter dimensions (E) 17.56 ± 1.3 μm (Fig. 3d and e, Table 1). The CL pollen grains were prolate spheroidal, with polar axis length 18.33 ± 1.82 μm and equatorial axis length 16.67 ± 2.02 μm (Fig. 3a and d, Table 1). The exine ornamentation of the CH pollen appeared smooth, whereas the surface of CL pollen was ornamented with granulating (Fig. 3c and f). CH and CL germination apertures diameter were 2.43 ± 0.06 μm and 2.68 ± 0.26 μm, respectively (Table 1).

**Table 1:** Characteristics of CH and CL pollen grain.

Types	Germination apertures dimensions (μm)	Polar axis lengths (P) (μm)	Equatorial axis length (E) (μm)	P/E ratio	Exine ornamentation
CH	2.43 ± 0.06b	18.33 ± 1.82b	16.67 ± 2.02ab	1.10	Smooth
CL	2.68 ± 0.26a	28.26 ± 1.31a	17.56 ± 1.3a	1.63	Granulate

### High-throughput sequencing analysis of small RNAs in *C*. *songorica* florets

To identify and characterize miRNAs involved in the differentiation of flower development in *C*. *songorica*, high-throughput sequencing was performed and four separate small RNA libraries constructed from the CH, CL_U, CL_M and CL_B flowers, respectively. RNA sequences of each library were annotated and classified as seven types including miRNA, rRNA, rRNAetc, snRNA, snoRNA, tRNA, and the unannotated (Table S1). After removing low-quality reads, 11.42, 10.78, 10.72 and 10.76 million cleans reads were generated from CH, CL_U, CL_M and CL_B, respectively; and a total of 1.22, 1.71, 2.01 and 1.44 million unique reads were generated from the four libraries. However, the ratio of miRNA was particularly low both in total reads and in unique reads. The length of small RNAs varied from 18 to 30 nt, and majority of them were concentrated in the 21 nt and 24 nt. The 24 nt sequence length was the most abundant. Moreover, the four libraries showed the similar trend (Fig. S3).

### Analysis of transcriptome sequences in *C*. *songorica*

All data from the raw sequence reads were deposited in the NCBI Bioproject with accession number PRJNA356791, biosample accession numbers SRS1846912 for CSCH and SRS1846913 for CSCL. The clean reads from each library were separately assembled using the Trinity de novo assembly program. In total, 145,196 contigs with an average length of 306 bp from the CSCL cDNA library and 153,487 contigs with an average length of 301 bp from the CSCH cDNA library were obtained (Table S2). We also obtained 74,782 unigenes from CSCL with a mean length of 584 bp and 79,145 unigenes from CSCH with a mean length of 582 bp (Table S2). Clustering of CSCL and CSCH generated 69,035 unigenes with an average length of 761 bp and an N50 length of 1217 bp.

### Identification of miRNAs and analysis of differential expression of miRNAs

Among the total miRNA sequences, 110, 104, 157 and 161 miRNAs (including known and novel miRNA) were expressed in the CH, CL_U, CL_M and CL_B flowers, respectively (Fig. S4a). The Venn diagram showed that a total of 51 miRNAs were shared by the four classes of flowers and 64 miRNAs were common to CL_U, CL_M and CL_B flowers (Fig. S4a). We also found that 36 miRNAs only were expressed in CH flower, 22 miRNAs only in CL_B flower, 56 miRNAs only in CL_M flower and 59 miRNAs only in CL_U flower.

Comparing to differential expression miRNAs in CH and CL, we found that 22, 56, 59 miRNAs was only expressed in CHvsCL_B, CHvsCL_M and CHvsCL_U, respectively; and 123 miRNAs were overlapped (Fig. S4b). In addition, other comparisons indicated that 106 differential expression miRNAs were common in CL_BvsCL_M, CL_BvsCL_U, CL_MvsCL_U and CHvsCL_U,CL_M,CL_B (CL_), 24 differential expression miRNAs expressed in CL_BvsCL_M, CL_BvsCL_U and CHvsCL_. Thirty-six differential expression miRNAs were identified from CHvsCL_ but not from other combinations. Sixty-one miRNAs were expressing in CL_BvsCL_U and CHvsCL_, and the same number miRNAs expressed in CL_BvsCL_M and CL_MvsCL_U (Fig. S4c).

**Figure 4:**
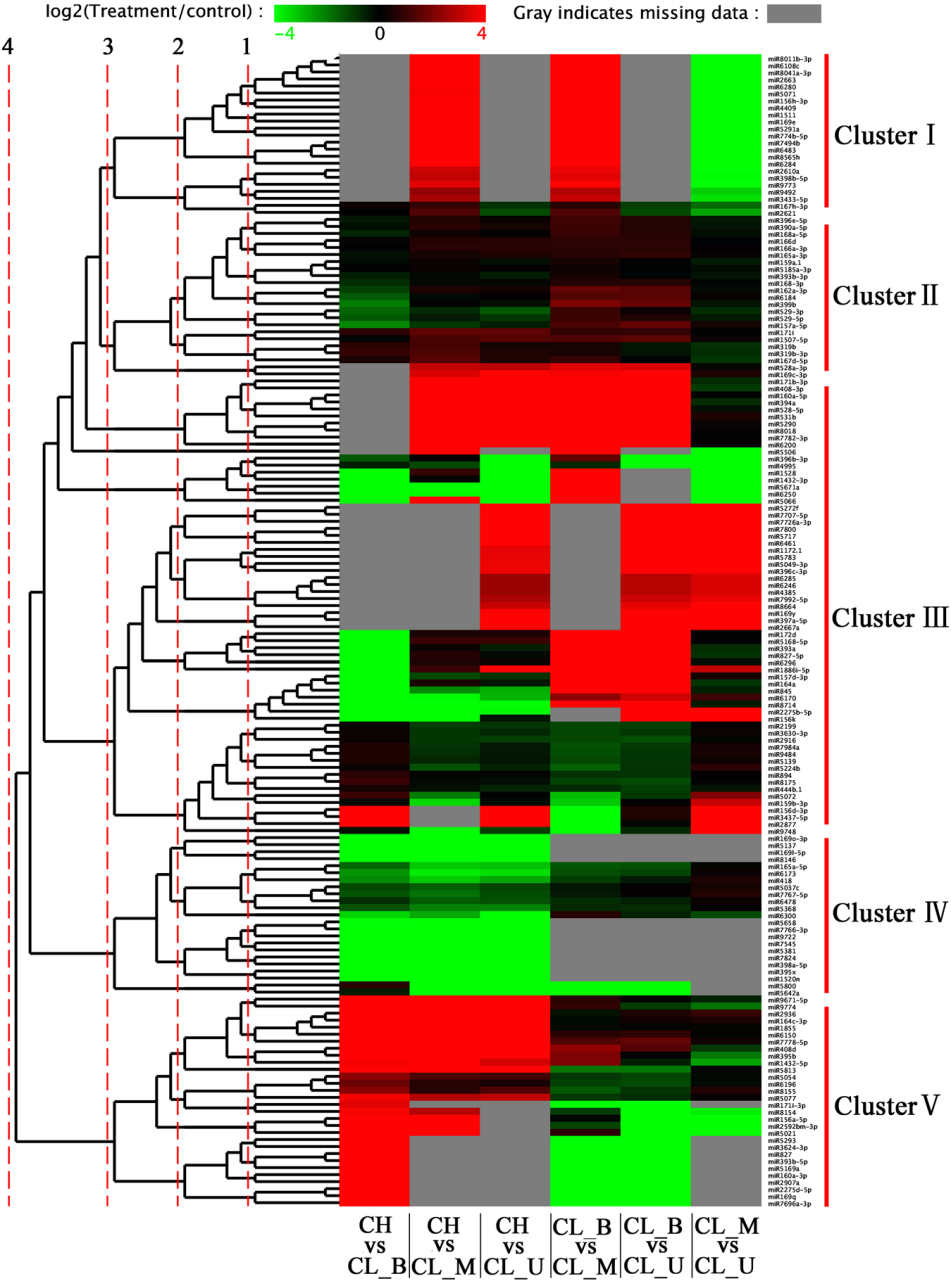
Expression pattern profiles of known differential expression miRNAs in different combinations. In the heat map representation, genes with similar expression patterns in the clones were clustered MeV_4_9_0. Expression clusters (including five classifications) are shown in the left and miRNA names are at the right. Color legend at top represents differential expression in microarray data.

**Figure 5:**
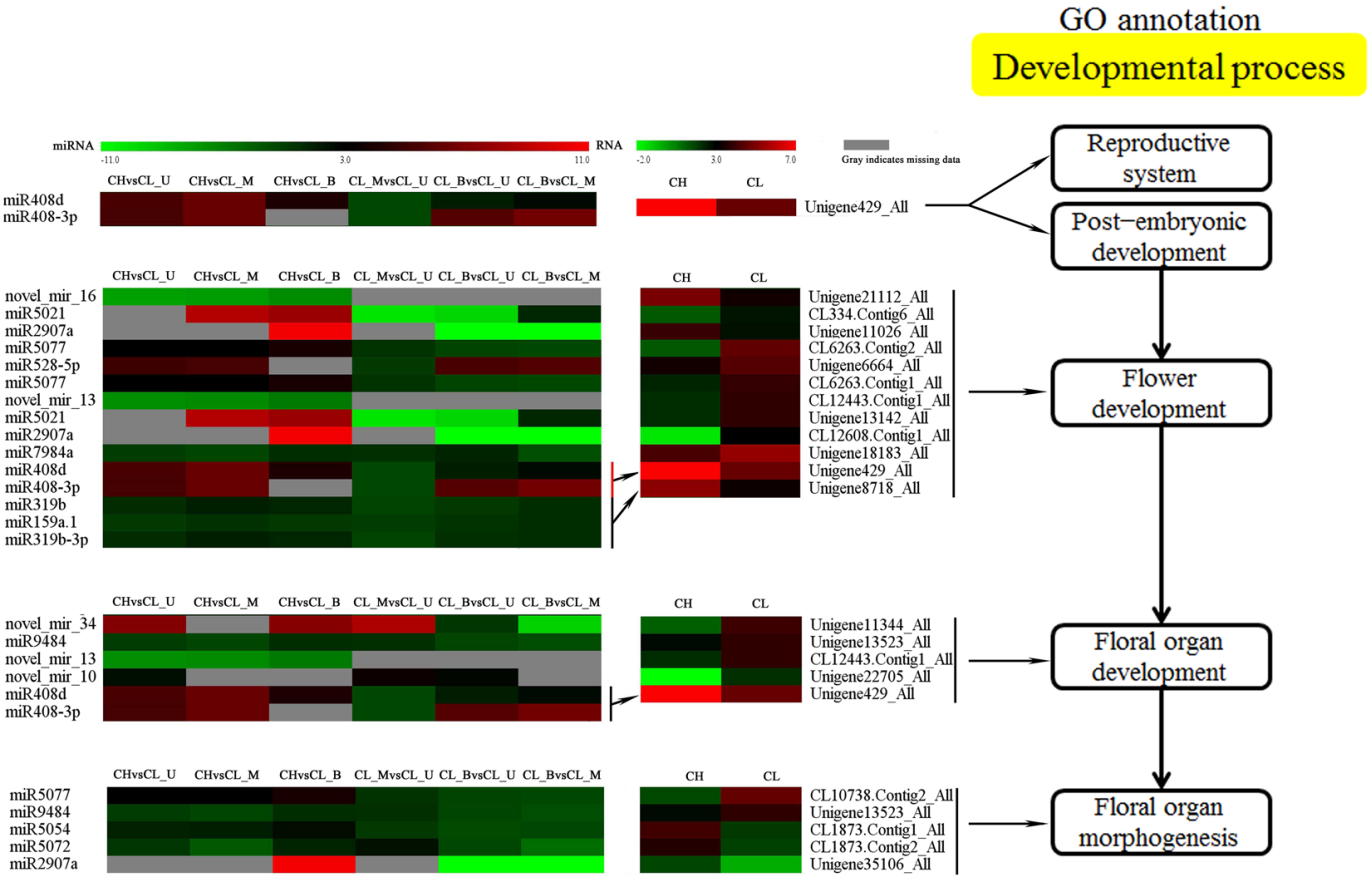
Expression patterns of miRNA-target genes pairs were speculated involved in flower development process. Expression patterns of differential expression miRNAs and their target genes were showing the left part and flower developmental process was displayed in the right part. The yellow box was a title of the flower developmental process.

Expression patterns of 164 miRNAs were determined using differential expression levels of each miRNA among all the pairwise comparisons of CH, CL_U, CL_M and CL_B. We created a heat map based on miRNA expression values, in which expression patterns of differential expression miRNAs were divided into five clusters (Fig. 4 and Table S3). As shown in Fig. 5, cluster I was distinct, miRNAs were highly expressed in CHvsCL_M and CL_BvsCL_M and lowly expressed in CL_MvsCL_U, but not expressed in CHvsCL_B, CHvsCL_U and CL_BvsCL_U, besides, miR156h-3p was up-regulated and quite high expression in CHvsCL_M and CL_BvsCL_M. The difference of miRNAs expression was very small among the six combinations (cluster II), particularly, miR159a.1 was highly expressed in CHvsCL_M and CL_BvsCL_M. Cluster III contained more than one third of the differentially expressed miRNAs. Among them, miR156k, miR156d-3p, miR159b-3p, miR172d exhibited a high expression in CL_BvsCL_U among the six combinations evaluated. However, miR156a-5p shown the highest expression in CHvsCL_B than in other combinations. Less than 17% of differentially expressed miRNAs exhibited a tiny expression in CHvsCL_B, CHvsCL_M and in CHvsCL_U (cluster IV). Thirteen of them showed no significant expression, and 8 of them were close to 0 in CL_BvsCL_M, CL_BvsCL_U and CL_MvsCL_U (cluster IV). In the cluster V, miRNAs were highly expressed in CHvsCL_B, but lowly expressed in CL_BvsCL_M and CL_BvsCL_U, and some of miRNAs didn’t expressed in CHvsCL_M, CHvsCL_U and CL_MvsCL_U (which means miRNAs only expressed in CL_B). The differential expression of 124 novel miRNAs was showed in Fig. S5 (Table S4).

### Potential target genes of miRNA prediction and annotation

The potential target genes of *C*. *songorica* miRNA were predicted by PsRobot. 1,657, 1,776, 1,868 and 1,407 potential target genes were identified and were corresponding to 78 miRNAs in CH flowers, 73 miRNAs in CL_B flowers, 99 miRNAs in CL_M, and to 90 miRNAs in CL_U, respectively (Table S5). To assess the transcriptome library of flower completeness and the annotation reliability, miRNA target genes were aligned to GO and KEGG database. In known miRNA target genes group, a total of 996 (CH), 1008 (CL_B), 1102 (CL_M) and 809 (CL_U) target genes were annotated and classified based on GO term (Fig. S6). In novel miRNA target genes, 289 (CH), 190 (CL_B), 420 (CL_M) and 425 (CL_U) target genes were annotated and classified based on GO term (Fig. S7). Thirty-one GO classes were observed in the known miRNA of the four constructed libraries (CH, CL_B, CL_M and CL_U flowers). 24, 18, 22 and 28 GO classes were investigated for novel miRNA in the respective libraries (Figs S6 and S7). The results suggested that ‘cell’ and ‘cell part’ were the largest proportion with regard to cell component category, ‘binding’ was the most frequent in the molecular function category and ‘metabolic process’ was the most highly represented groups under the biological process category both in known and in novel miRNA target genes (Figs S6 and S7). The target genes were annotated based on KEGG database to study biochemical pathways and we selected the top 20 most represented pathways to construct an enrichment plot. There are 100 miRNA target genes were enriched in ‘metabolic pathways’ (Fig. S8).

### Expression analysis of flower related miRNAs and their target genes based on GO annotation

Based on GO enrichment and function analysis and flower development process (Table S6), we constructed the processes for 17 miRNAs and 19 target genes, which were associated with different pathways (Fig. 5). For example, miR408d and miR408-3p targeted Unigene429, which is associated with reproductive system and post-embryonic development. Moreover, both the miRNAs and Unigene429 exhibited as fix-fold of differential expression in CH flower than in CL flowers. There are 14 miRNAs and 12 target genes associated with flower development. Obviously, novel_mir_13 and novel_mir_16 were expressed only in CH flowers but not in CL flowers. The expression level of their target genes, CL12443.Contig1 related to pollen development and Unigene21112 associated with *GLOBOSA* l gene, respectively, were higher in CL flowers than CH. miR319b, miR159a.1 and miR319b-3p targeted the same gene, Unigene8718. The expression level of miRNA was slightly higher in CH flowers than in CL flowers, and the expression pattern of the target gene displayed the same trend. Six miRNAs regulated 5 target genes that were related to floral organ development. Five miRNAs targeted five genes which were associated with floral organ morphogenesis pathway.

A total of 19 genes were targeted by 4 miR156 family members including miR156k, miR156a-5p, miR156d-3p and miR156h-3p. There were 14 genes that were targeted by the miR156a-5p which was significantly up-regulated (*p* < 0.001). From the 14 target genes, three genes were significantly down-regulated (*p* < 0.001), whereas 11 target genes were significantly up-regulated (*p* < 0.001). Besides, the target, CL1954.Contig2 response to high light intensity. Eleven targeted genes were shared by miR156k and miR156a-5p. A total of 11 genes were targeted by miR159a.1 including 8 genes that were significantly down-regulated (*p* < 10^−11^), and 3 significantly up-regulated (*p* < 10^−4^) genes. From the 11 genes, CL3996.Contig2 and CL11879.Contig4 were associated with flower development (Table S7). In general, the same miRNA could respond to different target genes with discrepant expression patterns and different pathways (Fig. 5). Furthermore, these results suggested that those miRNAs may mediate the level of gene expression to influence the morphogenesis of CH and CL flowers.

### The expression analysis of miRNAs and their targets by qRT-PCR validation

**Figure 6:**
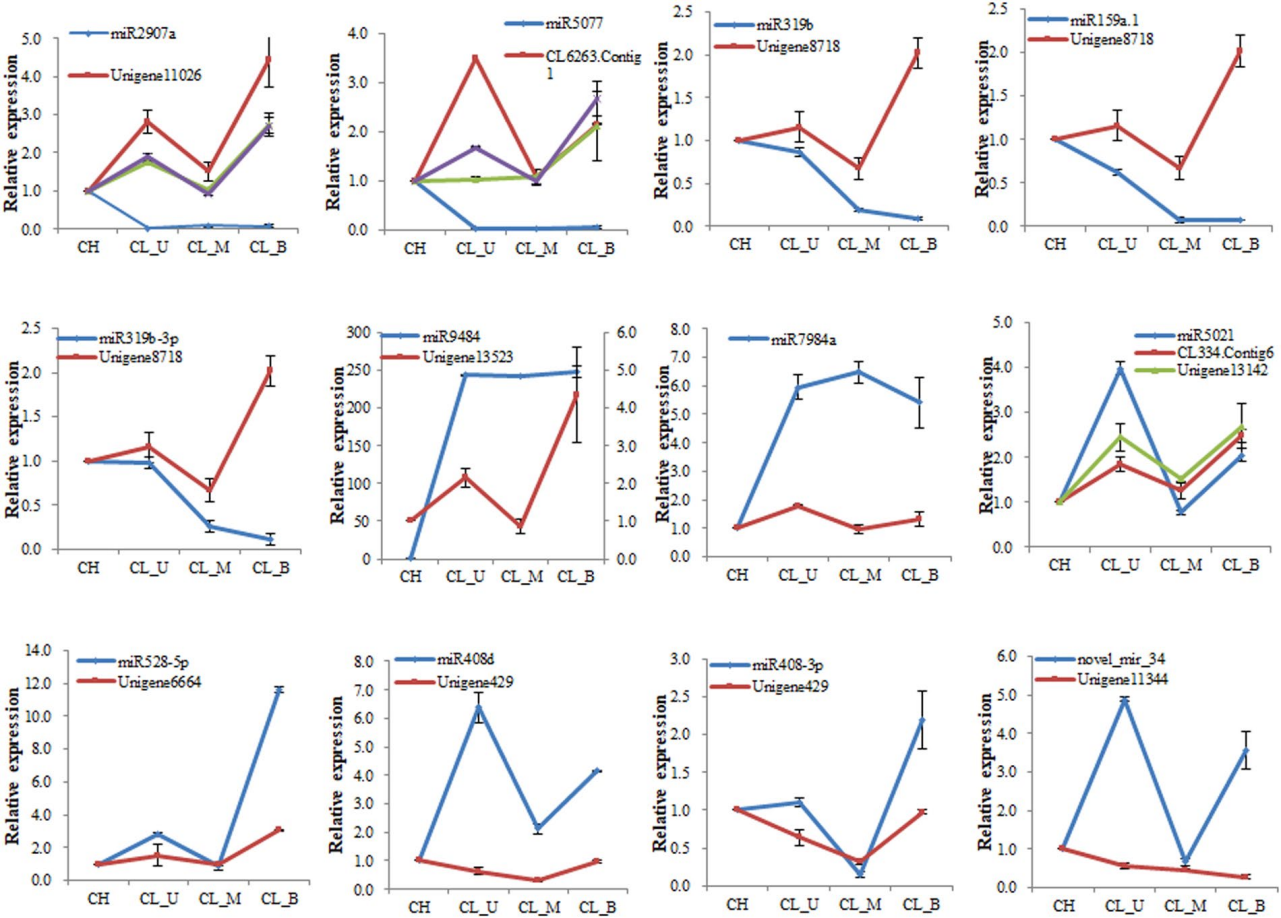
qRT-PCR validation of miRNAs and their target genes expression.

qRT-PCR was performed to validate the expression pattern of identified miRNAs and their target genes. Twelve miRNAs and their target genes were randomly selected to analyze the expression patterns in *C*. *songorica* (Fig. 6). Among the 12 miRNAs, miRNA-target pair in CH library was chosen as control. miRNA9484 was significantly expressed in three CL libraries but not expressed in CH library while the expression level of target gene was relatively low. However, the expression pattern of miRNA9484 was positively correlated with the target gene. miRNA7984a was highly expressed in the three CL libraries while the expression level of target gene Unigene18183 was very low (closed to 1), this miRNA-target pair presented a negative correlation. Furthermore, five miRNA-target pairs (miRNA including miR2907a, miRNA5077, miRNA319b, miRNA159a.1 and miRNA319b-3p) presented similar expression profiles as their respective miRNAs were down-regulated, while their target genes were up-regulated except some genes in CL_M library. miR528-5p, miR408d, miR408-3p and their target genes were displayed a positive correlation. However, among the 12 validated miRNAs, the expression trend of nine miRNAs and qRT-PCR results were similar with the sequencing results (Fig. S9). This was caused by the different sensitivity of high-throughput sequencing and the qRT-PCR detection method for specific miRNAs (Figs 4, 5 and 6).

### GO function analysis

**Figure 7:**
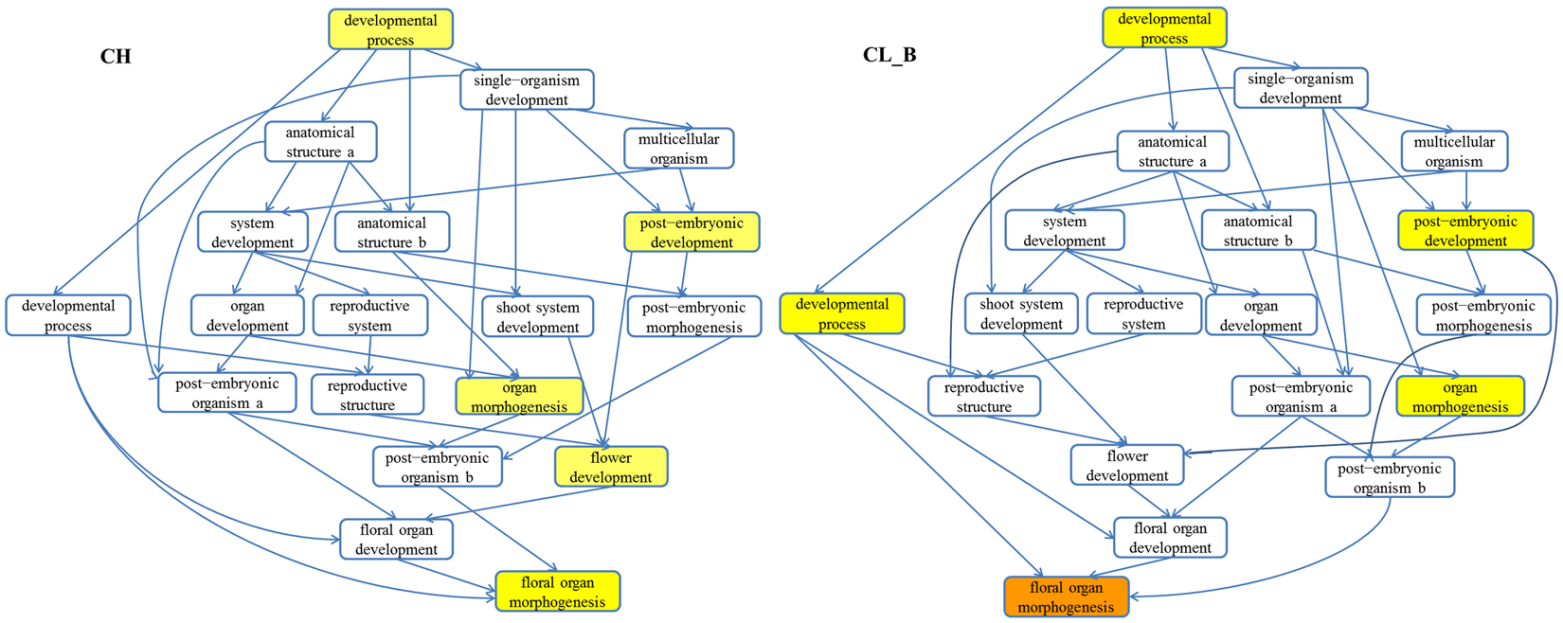
Enriched development processes involved by miRNAs and their target genes in CH and CL_B flower. Yellow colour (significant enrichment) represents P-value from 10^−2^ to 10^−1^.

GOrilla was performed to visualize development processes enrichment (a part of biological process)^31^. GO term ID of each process was matched with the gene which have been annotated based on GO term and have GO term ID. A total of 9 miRNAs and their 8 target genes were enriched to 19 development processes (Table S8). In CH and CL_B flower, several hubs were significantly enriched, including development process (GO:0032502), post-embryonic development (GO:0009791), organ morphogenesis (GO:0009887), flower development (GO:0009908) and floral organ morphogenesis (GO:0048444) (Figs 7 and S10).

**Figure 8:**
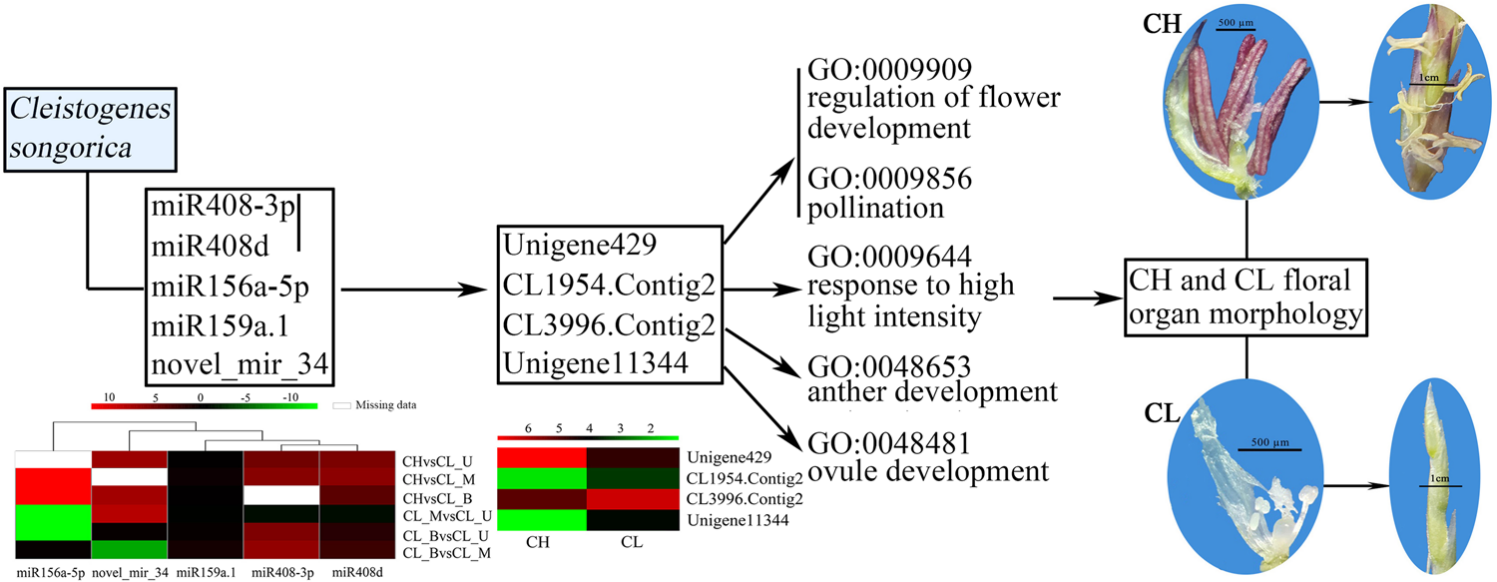
Four miRNA-target pairs were speculated regulated diverse floral organ development, which was based on GO annotation. The miRNA-target pairs were speculated to regulate CH and CL floral organ morphology because the expression of miRNAs and target genes was different between CH flower and CL flower. Heat-map under the miRNA were constructed based on miRNAs different expression level, the heat-map under gene were constructed based on the gene expression level.

Four pairs of miRNA-target were related to flower development based on GO function annotation which may regulate the CH and CL diverse floral organ morphology because of different expression results between CH and CL flowers (Fig. 8). The expression level of miRNA408-3p/miR408d was higher in CH flowers than in CL flowers. The target gene, Unigene 429 had a higher expression in CH flower than in CL flowers and was related to the regulation of flower development and pollination. Therefore, there was a positive correlation between miRNA408-3p/miR408d and Unigene 429 pair. However, the other three pairs (miR156a-5p-CL1954.Contig2, miR159a.1-CL3996.Contig2 and novel_mir_34-Unigene11344) showed a negative correlation; miRNAs had a high expression in CH flower while the expression of their respective target genes was higher in CL flowers.

### miRNAs and their targets verified by *C*. *songorica* reference genome data

The predicted miRNA and their targets were further evaluated with *C*. *songorica* reference genome for any false-positive predictions. Some of the identified flower related miRNAs were aligned with same miRNAs family (like miR408, miR156, miR159) in reference genomes, and some of them (miR156k-mdm_miR156t, miR159a.1-mdm_miR159a and miR159b-3p-mdm_miR159b) expression trend was similar (Table S11). The targets of flower-related differential expression miRNAs were all aligned to *C*. *songorica* genes (Table S12). The result indicated that the targets were fragment or entire of *C*. *songorica* genes. Ten of miRNA targets and *C*. *songorica* genes alignments were shown in the Figures S11–20.

## Discussion

This is the first time to analyze the natural dimorphic cleistogamous plant using miRNA sequencing combined with floral morphological traits of CH and CL to understand molecular mechanisms of floral differentiation and development. Dimorphic plant could produce both CH and CL flowers during the whole growth cycle even under different environmental conditions. Previous studies played more attention to understand ecological factors under CH and CL breeding system, such as water effect, length of photoperiod, level of temperature and availability of soil fertility. A similar conclusion was that plant produced dimorphic flower to adapt evolution^32–34^. However, research also suggested that gene expression regulated flower development to response to environmental changing^10^. In our study, dimorphic plant, *C*. *songorica* CH flowers and three parts of CL flowers were surveyed for morphological traits of floral organ and molecular mechanisms.

Flower development plays vital roles in the life cycles of *C*. *songorica*. In this study, the size of small RNAs is from 21 nt to 24 nt which is consistent with previous reports, such as *Petunia axillaris* flower buds study^24^, and *Camellia* floral organs study^35^, etc. Here, all the potential target genes were classified based on GO, KEGG and COG annotations which provided basal information for further studies on flowering-associated miRNAs in *C*. *songorica*.

Previous studies showed that miRNAs are critical during flower development^35–37^, particularly in CL flower growth^7^. All differentially expressed miRNAs were clustered, and expression levels of some miRNAs and their targets was significantly different in our study. We speculated that the difference in the expression of flower development related miRNA-target pairs could give rise to the phenotype difference of CH and CL flowers, as previously indicated that over-expressing of miR171c and its target gene *scl6*, altered flower structure^38^. miRNA39, also known as miR171, is accumulated predominantly in inflorescence tissues and interacted with the target genes to encode several putative transcription factors in *Arabidopsis*^39^. In this study, perhaps three miR171 members co-regulated some genes and contributed to the observed different morphologies of CH and CL flowers as they have different expression patterns in CH and CL flowers. A single nucleotide mutation of miR172 targeting site led to failure of lodicules development which produced a CL phenotype^7^. Similar result was found in *C*. *songorica* as miR172d was down-regulated in CH flower, but up-regulated in CL flower and dimorphic flowers were formed. OsmiR156b and OsmiR156h over-expression can cause some variations during flowering, including delayed flowering time, decreased panicle and reduced stature in rice^40^. Our results are somehow consistent with the earlier reports as it showed that miR156a-5p and miR156d-3p are significantly up-regulated resulting into smaller panicle in CL flowers. A previous study showed that the expression of miRNA396 targeted 9 GRFs mediates a number of carpel and the pistil development in *Arabidopsis*^41^. Overexpression of AtmiR159 resulted in anthers defects or male sterility in *Arabidopsis*^42^ and it was reported miR159 was involved in controlling flowering time^43^. In our study, there is a great discrepancy in anther shape and pistil size between CH and CL on the morphological side. Therefore, miRNAs may regulate flower development of *C*. *songorica* and cause floret divergence between CH and CL. However, further functional characterizations such as genetic manipulation of *C*. *songorica* CH and CL florets should be performed to verify these leads.

The early floral primordia of *C*. *songorica* CH and CL flowers were similar, whereas the morphology and color of CH and CL floral organs were discrepant with the flower developing (Fig. S2). Classic ABC/ABCE model was proposed as a mechanism to explain floral organ formation in angiosperms, where three or four classes of genes, or their combinations controlled organ formation in each whorl^44–47^. The ABC model was also valid in monocotyledonous plants, such as rice^48,49^. The A class gene *AP1* regulated the development of sepals and petals which were replaced by lemma, palea and lodicules in rice^50^. In *C*. *songocira*, the lemma length of CH was slightly larger than that of CL. In addition, novel_mir_34 targeted Unigene11344 highly expressed in CH library was involved in regulating ovule development. The expression difference of novel_mir_34-Unigene11344 may regulate lemma discrepancy in CH and CL flower. B-class floral homeotic genes control petals and stamens development. Our results showed that anther size of CH was larger compared to CL. Furthermore, the expression of CL3996.Contig2, involved in anther development, was significantly up-regulated in CL library, which was targeted by miR159a.1 (significantly down-regulated in CL_U and CL_B library). A previous study has shown that over-expressed miR159 (negative regulated GAMY expression) exhibited significantly late flowering in transgenic gloxinia plants and suppression of miR159 caused a conversion of petals to sepals in a few transgenic plants^51^. Thus, the results suggested that the negative interaction between miR159a.1 and its target expression may suppress the CL flowering. However, the size of pollen and filament of *C*. *songorica* CL flower was far larger than that of CH flower. Meanwhile, the miR408-3p/miR408d-Unigene429 positively-regulated pair was associated with pollen development. Pollen volume and the time taken for pollen tubes to reach the ovary were both positively related to pistil length. And in general, the larger pollens, with more rich nutrients stored, facilitate the greater growth rates of the pollen-tube^52–54^. Therefore, as *C*. *songorca* CL pollens are bigger and filaments are longer, CL seed setting rate and thousand seed mass were higher than that of CH. In CH flower, the lemma was pushed by swelling lodicule which was the key factor to control flower opening^55,56^. Conservation of the expression in lodicules is in perfect agreement with B-function of the *GLO*-like genes in grasses^57^. In our study, novel_mir_16 was only expressed in CH library, and its target Unigene21112 (associated with *GLOBOSA*) was highly expressed in CH library. Morphologically, the lodicules of CL were very small and nearly atrophied while the length and thickness of CH lodicules were apparently large. Similar results were seen in barley^7^ where the size of floral organs lodicule decreases in CL. The results may explain CH flowers are more readily to open than CL flowers. Further investigations are deserved on CL related genes and their possible relations to the genes involved in the ABC model.

In conclusion, a comprehensive study on floral organ growth and development, and miRNAs-target genes related to CL and CH flower on *C*. *songorica* have been conducted. The phenotypic differences of CL and CH floral organs have been recorded. Strikingly, the pollen grain and ovary of a CL flower were larger than that of CH flower. We have identified 17 miRNAs and 19 target genes that are associated with flower development. The pairs of miRNA and its target gene, such as miR159a.1-CL3996.Contig2, miR156a-5p-CL1954.Contig2 and miR408-3p/miR408d-Unigene429, have highly expression level, were highly related to flower development based on the GO analyses and in agreement with the other published results. Considering the unique flower structures in *C*. *songorica*, this study provides useful information to elucidate the roles of miRNA and its target genes in the differential development of cleistogamy and chasmogamy in this species.

## Methods

### Plant materials

*C*. *songorica* materials used in this study were grown in the field of Yuzhong (104°12′ N, 35°85′ E; Yuzhong county, Lanzhou, China). The whole flower structure of *C*. *songorica* includes CH flowers (top spike) and CL flowers (below flowers, usually including 9 nodes flowers). Therefore, we divided the flowers along the individual internodes into four sections: CH spike, CL_U nodes (upper nodes, from 7 to 9), CL_M nodes (middle nodes, from 4 to 6) and CL_B nodes (bottom nodes, from 1 and 3 nodes).

Each sample was collected from thirty tillers. For the CL_B sample, we chose the tillers which only had three nodes, and then we took florets out from the three sheaths. For the CL_M sample, we selected the tillers which have five or six nodes, and then we took florets from the upper two or three nodes. For the CL_U sample, we selected the tillers which have 9 nodes, and then we took florets from the upper two or three nodes. All the samples were collected from fresh plants and immediately put in liquid nitrogen and stored in the −80 °C refrigerator.

### Seed traits at different positions

Seeds were collected from the field which consisted of four replicates: 6 m^2^ (3 by 2) for each plot^58^. Thirty randomly selected undamaged fertile tillers were individually collected from each plot in September and air-dried to 8 to 10% moisture. The CH, CL_U, CL_M and CL_B spikelet length, seed number and total seed mass were recorded. Thirty fertile tillers were randomly selected in each plot for measuring traits. The thousand seed mass was calculated based on the average mass of the four replicates, with each replicate of 100 seeds.

### Floret morphology of different development stages and floral organ observation

CH and CL flowers (lemma was stripped) at different development stages, were observed under a binocular dissecting microscope (SZ2-ILST, Olympus Corporation, Tokyo Japan). The stages at spikelet primordium, green anther, yellow anther and purple anther were photographed. At the green anther stage, flower organs, like lemma, anthers, filaments, stigma and stamens were investigated under the dissecting microscope, their length was measured and their appearance was photographed.

The morphology of florets was observed under the dissecting microscope. Thirty flowers were observed in each type (CH and CL). Meanwhile, the flowers were embedded in neutral gum, cross-cutting serially into 30 μm thickness, and stained with dyes of toluidine blue. The images were captured using a Nikon DXM 1200 color camera attached to a Nikon microphot-FX microscope system with ACT-1 software (Nikon, Japan).

### Scanning electron microscopy (SEM) observation and morphometric analysis of pollen

Anthers were taken from fresh flower and observed under microscope at purple anther stage. Pollen grains were transferred immediately to double-sided adhesive tape on the aluminum stubs. Before examining the collected pollen grains, they were coated with gold. For SEM observation, samples were coated with gold using a JFC-1100E Ion Sputter manufactured by JEOL. Micromorphological structures of pollen grains were observed and photographed by means of Hitachi S-4700 and Philips XL 20 scanning electron microscopes (Philips, Netherlands).

### RNA extraction, small RNA and mRNA library construction and sequencing

Total RNA was extracted from fresh flower (CH, CL_U, CL_M and CL_B flower) using the RNAprep pure Plant RNA Purification Kit (Tiangen Biotech., Beijing, China) according to the manufacturer’s instructions. The quality of total RNA was checked using a NanoDrop Spectrometer (ND-1000, USA) and gel electrophoresis.

Four small RNA libraries were constructed and sequencing by BGI Tech (Shenzhen, China). Briefly, PAGE gel was used to separate RNA segments to different size, 5′ and 3′ adaptors were ligated sequentially to small RNA, and then reverse transcription system was performed. Agilent Bioanalyzer 2100 system and ABI StepOnePlus Real-Time PCR System were used to assess the quality and yield of libraries. Those libraries were sequenced on Illumina HiSeq.2000 platform (San Diego, CA, USA), and 50 bp single-end reads were generated.

A total of 3 µg RNA per sample was used as input material for the RNA sample preparations. After the quality of the total extracted RNA was checked, high quality total RNA was sent to the Beijing Genomics Institute (BGI, Shenzhen, China) for cDNA library construction and sequencing based on the Illumina HiSeq 2000 platform.

### Bioinformatics analysis of small RNA sequences, miRNA target genes prediction and annotation

The raw sequences from HiSeq sequencing were first processed to removing the low quality tags and 5′ adaptor contaminants to obtain reliable clean reads. Then the clean reads were filtered to get rid of the reads either <18nt or >30nt. Tags were aligned to Genbank (http://www.ncbi.nlm.nih.gov/GenBank/) database with blast or bowtie. rRNA, tRNA, snRNA, snoRNA, repeat associated sRNA and degraded tags of exon or intron were screened and removed. Conserved miRNAs were identified through a Blastn search against the miRNA database, miRBase 19.0 (http://www.mirbase.org/). The reads, not being annotated to any known categories, were used to predict novel miRNAs by the miRNA prediction software Mireap (http://sourceforge.net/projects/mireap/), by aligning with *C*. *songorica* transcriptome data (PRJNA356791). Novel miRNA precursors were also predicted using the same data set.

The potential target genes of *C*. *songorica* miRNA were predicted using PsRobot (http://omicslab.genetics.ac.cn/psRobot/), and those predicted target genes were employed to annotate functions and pathways. Numerous target sequences were assigned to GO enrichment and KEGG pathway to obtain a clear biological information map of the miRNAs-dependent key biological processes. Web Gene Ontology Annotation Plotting (http://wego.genomics.org.cn/cgi-bin/wego/index.pl) were performed to analyze the miRNA target genes classification based on their associated cellular component, molecular function and biological process.

### Identification miRNAs and their target genes involved in flower development

The differential miRNAs were significantly expressed with larger than 2-fold changes and q-value less than 0.01. According to the miRNA and its candidate target genes, we conduct Gene Ontology enrichment analysis based on the set of target genes. miRNAs were matched with the flower-related genes to identify the flower-related miRNA. All *C*. *songorica* target genes have been annotated by the Gene Ontology database. In total of 19 target genes associated with controlling flowering process were identified based on GO term (GO:0032502 and GO:0003006 developmental process, GO:0061458 and GO:0048608 reproductive system, GO:0009887 organ morphogenesis, GO:0009908 flower development, GO:0048437 floral organ development, GO:0048444 floral organ morphogenesis, etc., http://amigo.geneontology.org/). From all the 19 sequences obtained, each sequence was used as a query sequence to check by using the online Blast (Nucleotide BLAST) (https://blast.ncbi.nlm.nih.gov/Blast.cgi). We searched the flower related sequences from the significant alignments (Query cover surpassed 90%). If the gene meets the criteria as what we mentioned above, we would identify the candidate genes related to flower in *C*. *songorica*.

### qRT-PCR of miRNAs and the target genes

qRT-PCR was performed to validate the identified miRNAs expression pattern and analyze the correlation between miRNAs and their targets. Total RNAs of CH, CL_U, CL_M and CL_B florets were reversely transcribed to cDNA, according to the manufacturer’s instructions of miRcute miRNA First-Strand Synthesis Kit (Sangon, Shanghai, China) and Mir-X™ miRNA qRT-PCR SYBR^®^ Kit (Takara, Dalian, China). Real-Time PCR System was performed according to manufacturer’s protocol of SYBR Premix Ex TaqTM and SYBR Green (TaKaRa, Dalian, China). miRNA primer was designed by Sangon Biotech company (Shanghai, China) (Table S9) and primers of target genes were designed using Primier 6 software (Table S10). All qRT-PCR reactions were performed on an Applied biosystems 7500 Real-Time PCR System. Three biological replicates for each sample were used and three technical replicates in 96-well plates were also used. The reactions were performed in a volume of 20 µL containing 2 µL of cDNA, 10 µL of SYBR Green PCR Master Mix (Applied Biosystems), 1 µL of each primer and 6 µL of ddH_2_O. miRNA and the target genes PCR reaction procedure were executed as follows: 10 min at 95 °C for DNA polymerase activation, followed by 40 cycles of 95 °C for 15 s and 60 °C for 1 min. Relative expression levels of miRNAs and their target genes were based on comparative *Ct* method while experimental data were normalized using U6 with the 2^−ΔΔCt^ method. Standard errors among three replicates were calculated.

### Verification of miRNAs and their targets with an in-house *C*. *songorica* reference genome

The clean reads of miRNA and RNA-seq were mapped to an in-house *C*. *songorica* reference genome (data were not published yet) in the BMK Cloud server. Then, we re-analyzed data using the software package of RNA-seq and small RNA in the BMK Cloud server and got genes and mdm-miRNAs. Flower related miRNAs were aligned to all mdm-miRNAs (from *C*. *songorica* reference genome) to identify non-false positive miRNA family, with the similarity >90%. The target transcript sequences were searched against whole *C*. *songorica* genes database by using local BLAST with e-value < 10^−5^.

## Electronic supplementary material

Supplementary Information

Dataset 3

Dataset 4
