# Supplementary material for: Analysis of microRNA reveals cleistogamous and chasmogamous floret divergence in dimorphic plant
Source: Sci Rep. 2018 Apr 19;8:6287. doi: 10.1038/s41598-018-24477-x (PMC5908857; doi:10.1038/s41598-018-24477-x)
Supplement: Supplementary file 1 — Supplementary Information [file 41598_2018_24477_MOESM1_ESM.docx]

**Analysis of microRNA reveals cleistogamous and chasmogamous floret divergence in dimorphic plant**

**Fan Wu**^1 ¶^**, Daiyu Zhang**^1 ¶^**, Blaise Pascal** **Muvunyi**^1^**, Qi Yan**^1^**, Yufei Zhang**^1^**, Zhuanzhuan Yan**^1^**, Mingshu Cao**^2^**, Yanrong Wang**^1^ **and Jiyu Zhang**^1^ *****

^1^. State Key Laboratory of Grassland Agro-ecosystems, College of Pastoral Agriculture Science and Technology, Lanzhou University, Lanzhou, China

^2^. AgResearch Limited, Grassland Research Centre, Palmerston North 4442, New Zealand

^¶^These authors contributed equally to this work.

***** Correspondence: [zhangjy@lzu.edu.cn](mailto:zhangjy@lzu.edu.cn); Tel.: +86-931-891-4051

**Supplemental Figure S1-S20**

**Supplemental Table S1-S12**


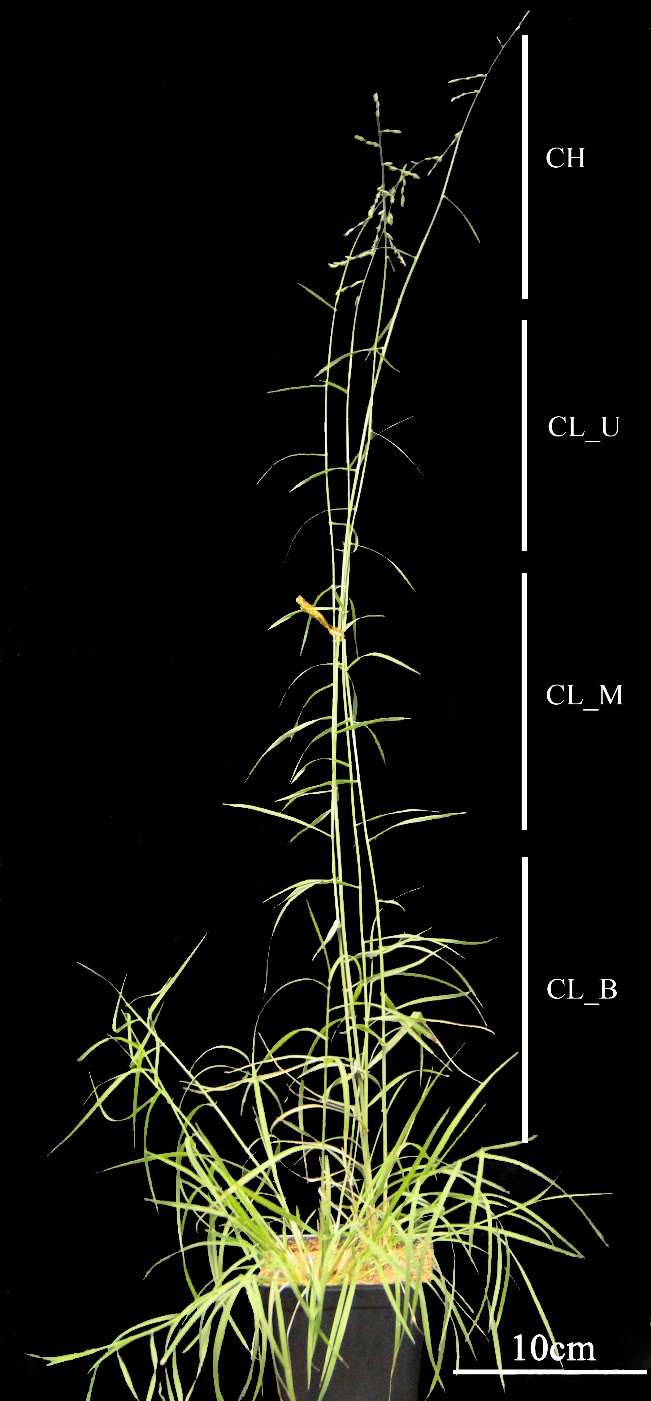


**Supplementary Fig. S1: The whole plant of *C. songorica*.**

**
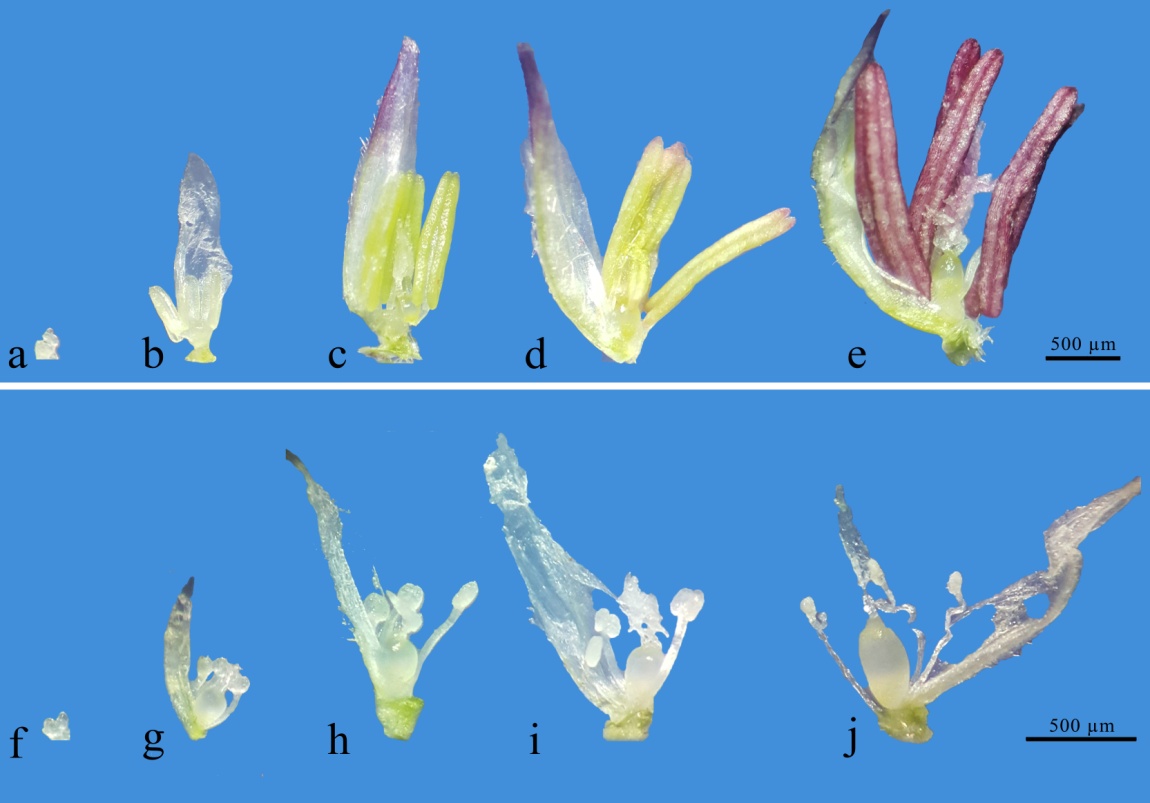
**

**Supplementary Fig. S2: Different stages of *C. songorica* floral development*.*** a-e are five stages of CH flower, they are floret primordium stage, white anther stage, green anther stage, yellow anther stage and purple anther stage, respectively. f-j are CL flowers five stages. Flower is developed from primordium and the colour of different stages CL floret was always white. All floret were observed under the dissecting microscope.


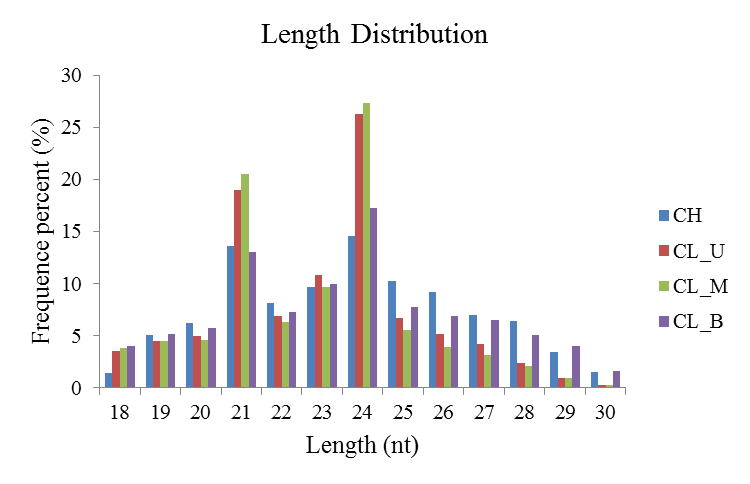


**Supplementary Fig. S3: Size distribution of small RNAs sequences in the four libraries of *C. songorica* floret**


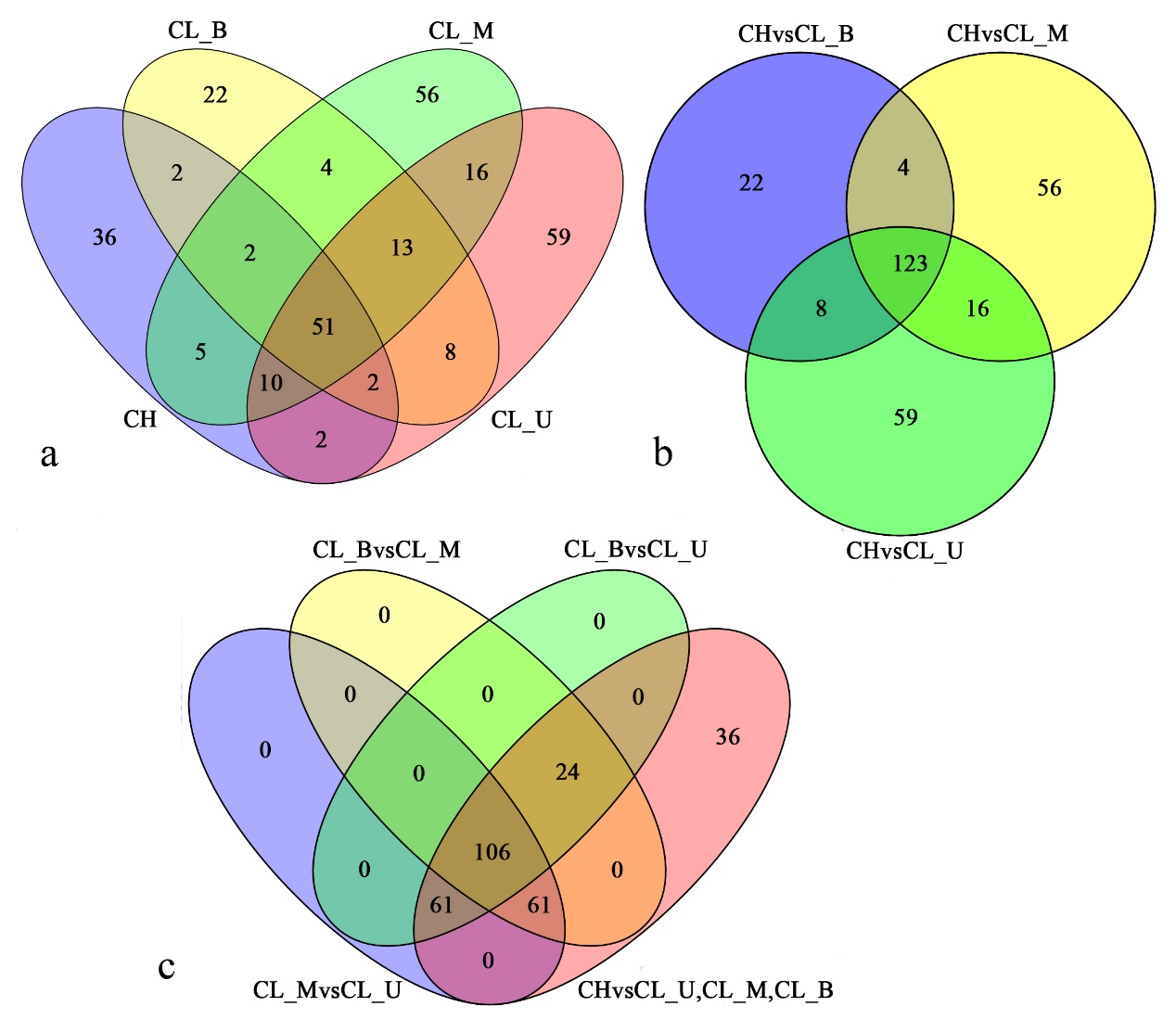


**Fig. S4: Distribution of *C. songorica* four positions flower miRNAs**. a, Common and special miRNAs from four libraries. b and c, Venn diagram of differential expression miRNAs from different combinations.

**
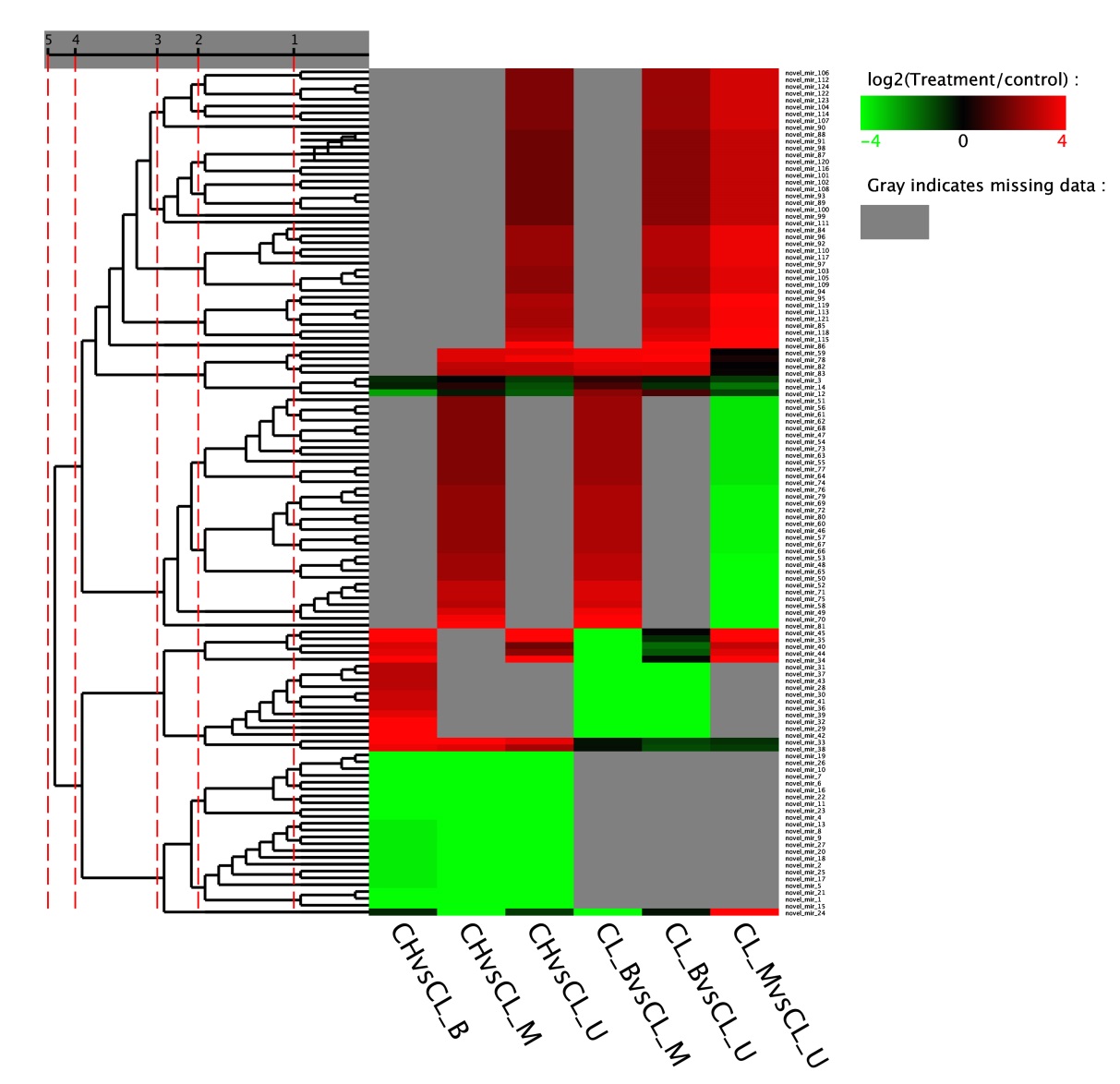
**

**Supplementary Fig. S5: Expression pattern profiles of novel differential expression miRNAs in different combinations.** In the heat map representation, genes with similar expression patterns in the clones were clustered MeV_4_9_0. Expression clusters (including five classifications) are shown in the left and miRNA names are at the right. Color legend at right represents differential expression in microarray data.


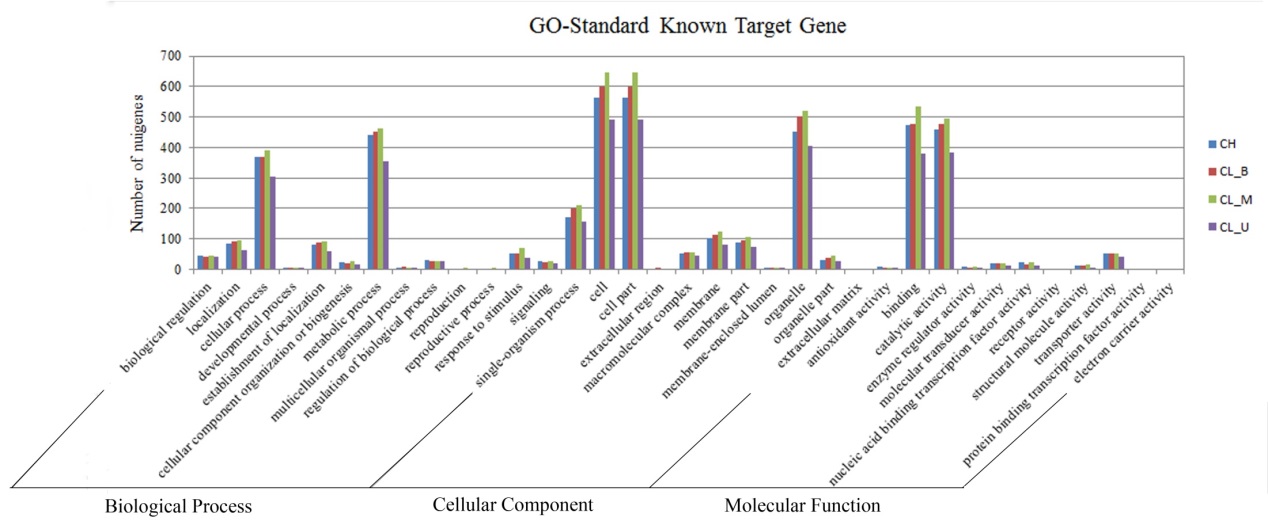


**Supplementary Fig. S6: GO analysis for known miRNA targets from *Cleistogenes songorica* according to the Gene Ontology (GO) programme.** The results are summarized under three main GO categories: BP-biological process, CC-cellular component and MF-molecular function.

**
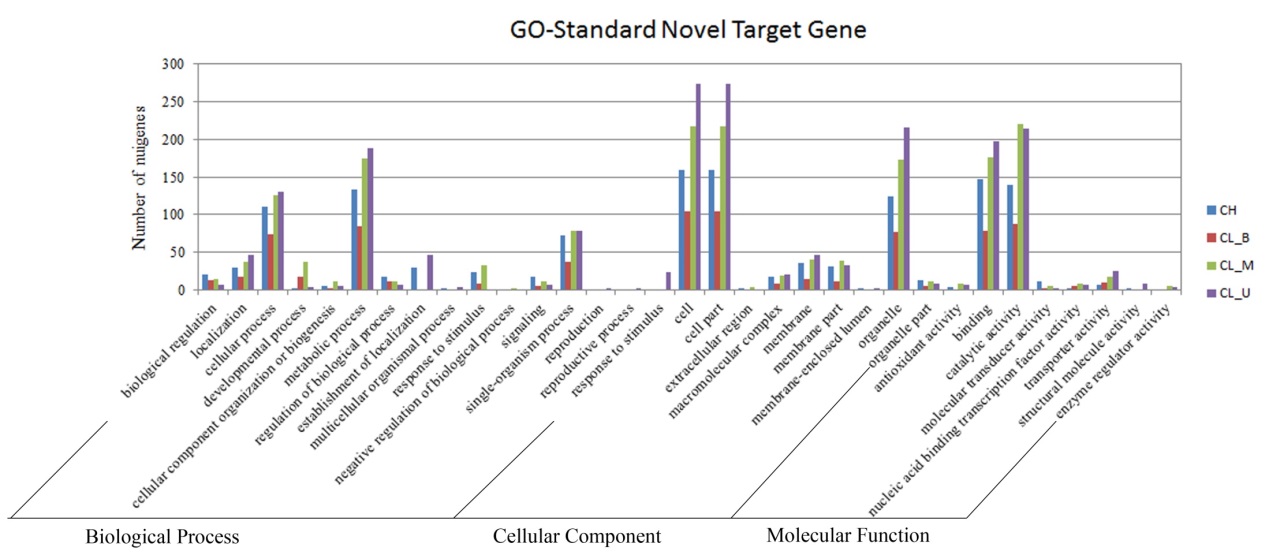
**

**Supplementary Fig. S7: GO analysis for novel miRNA targets from *Cleistogenes songorica* according to the Gene Ontology (GO) programme.** **Gene ontology (GO)Gene ontology (GO) classification of novel miRNA target genes.** The results are summarized under three main GO categories: BP-biological process, CC-cellular component and MF-molecular function.

**
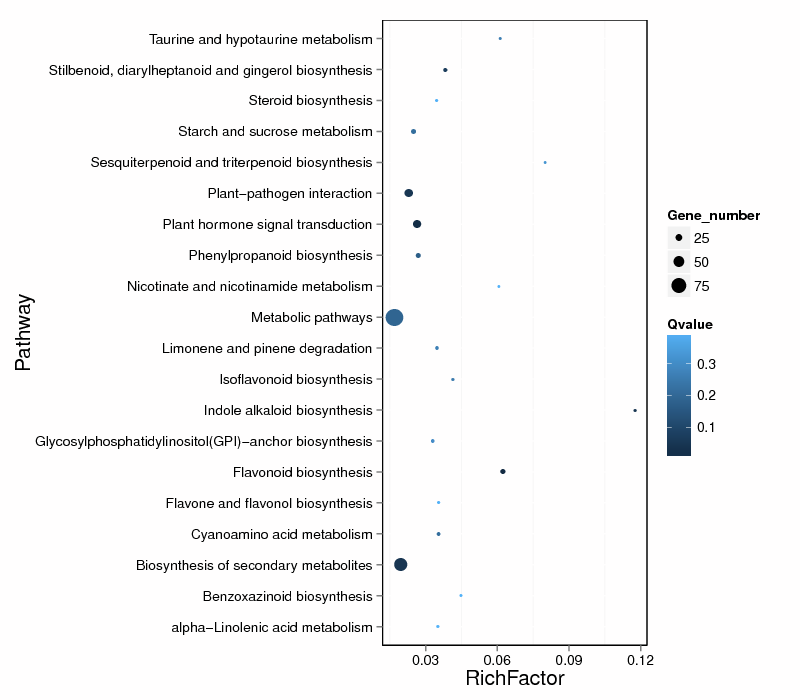
**

**Supplementary Fig. S8: The top of 20 pathways assignment (CHvsCL_U,CL_M,CL_B enrichment) based on KEGG database.**


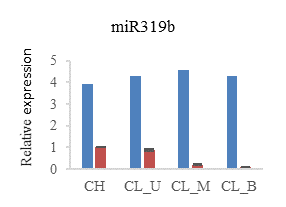

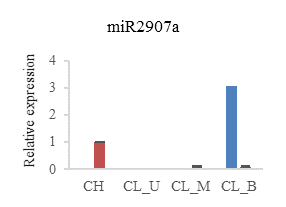

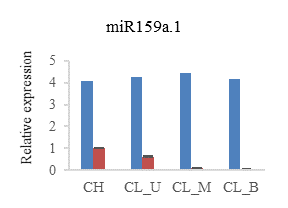

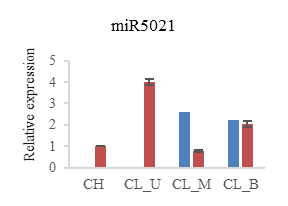

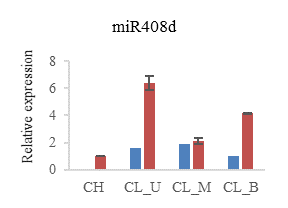

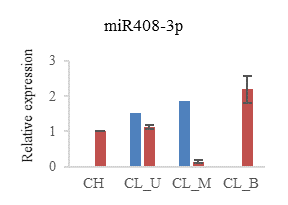

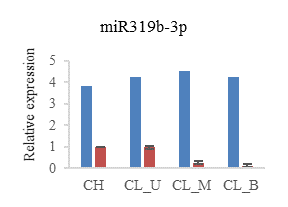

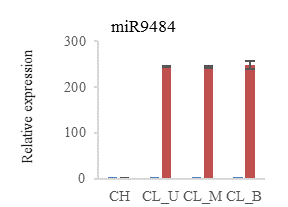

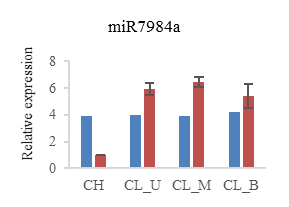

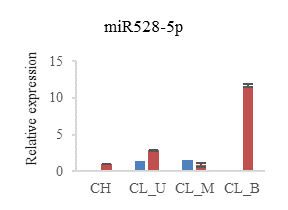

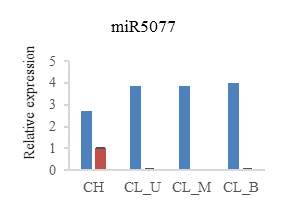

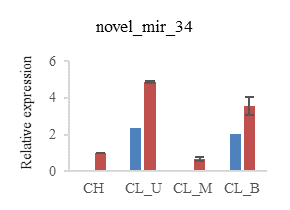


**Supplementary Fig. S9: Expression profiles of twelve miRNA.** The blue bar graph represented miRNA sequencing results, the value of y-axis was log_10_(TPM value). The red bar graph indicated the q-PCR results, data are mean ± SD from three biological replicates.


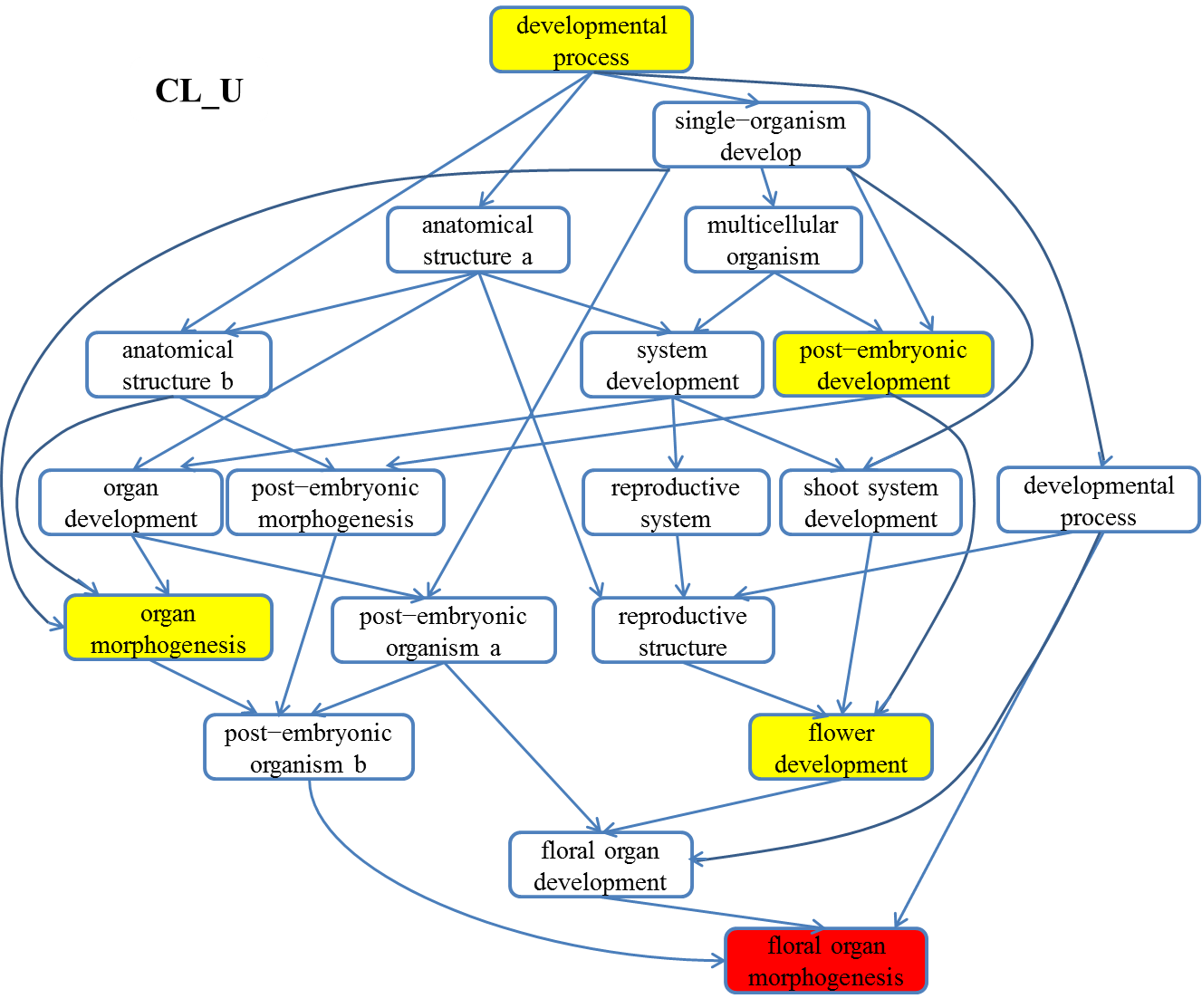


**Supplementary Fig. S10: Enriched development processes involved by miRNAs and their target genes in CL_U flower.** Yellow colour (significant enrichment) represents P-value from 10^-2^ to 10^-1^.

Alignments of target genes and C.songorica genes.

**Supplementary Fig. S11: The alignment of target gene (upper), CL6263.Contig1 and *C.songorica gene* (under), Cs1Chr10017715.**


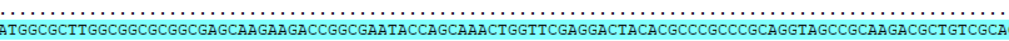


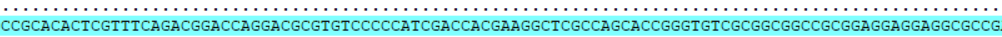


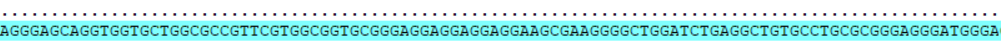


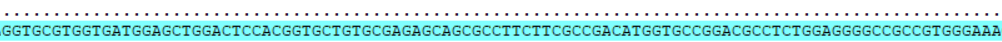


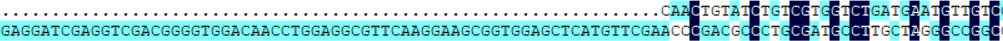


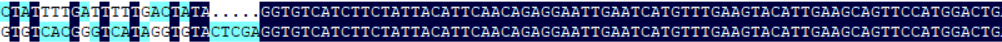


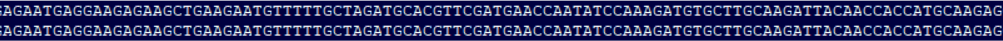


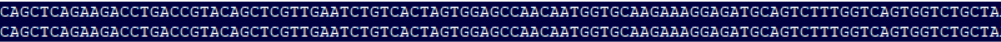


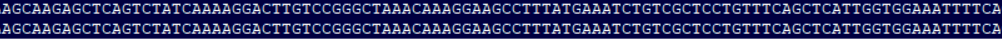


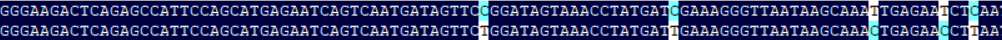


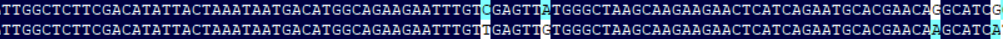


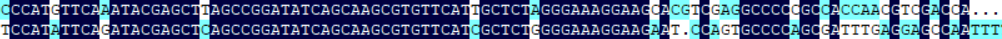


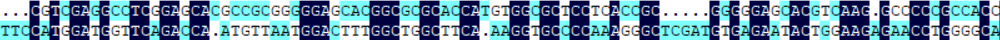


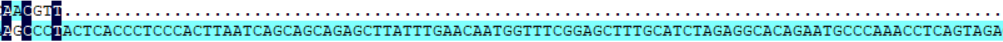


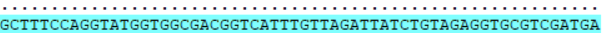


**Supplementary Fig. S12: The alignment of target gene (upper), CL6263.Contig2 and *C.songorica gene* (under), Cs1Chr10017715.**


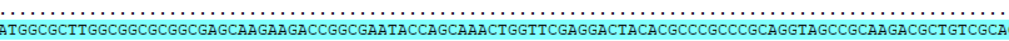


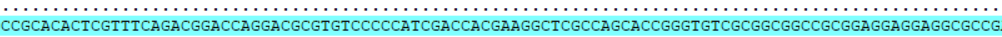


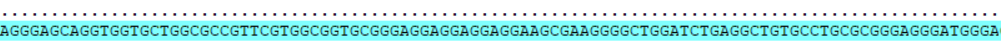


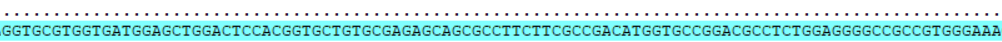


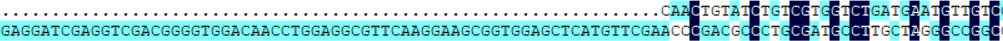


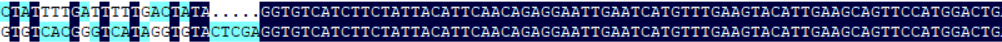


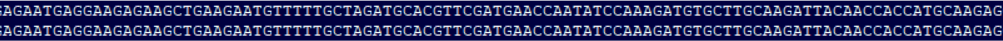


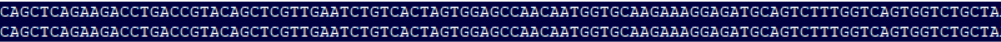


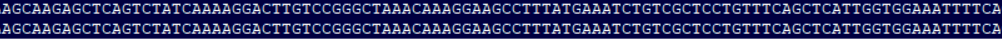


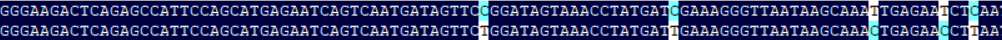


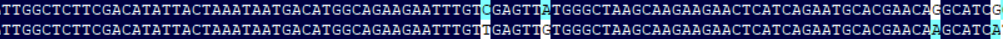


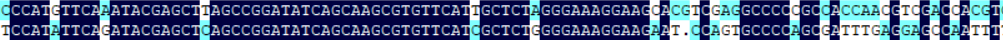


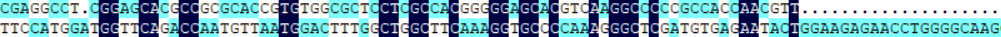


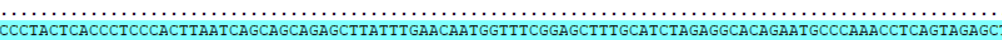


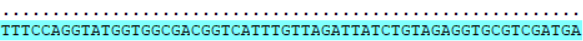


**Supplementary Fig. S13: The alignment of target gene (upper), Unigene6664_All and *C.songorica gene* (under), Cs3Chr10014223.**


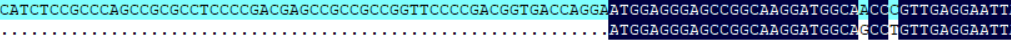


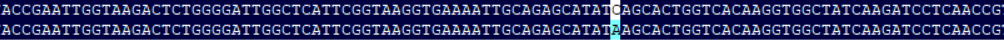


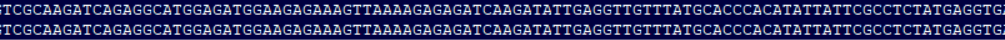


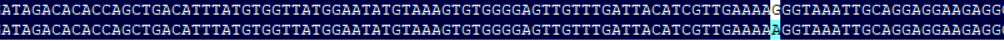


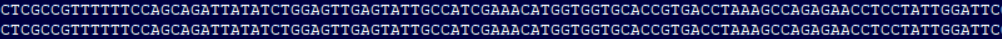


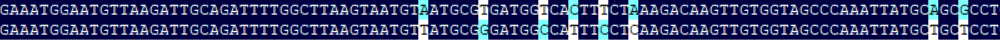


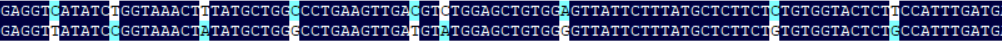


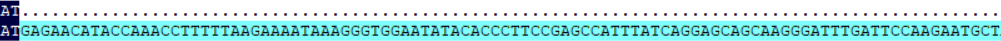


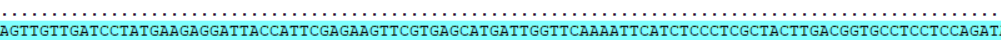


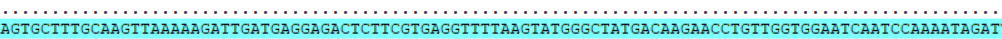


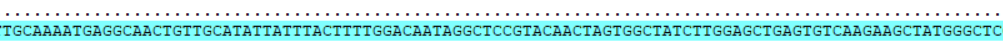


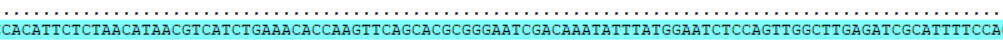


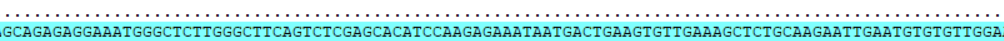


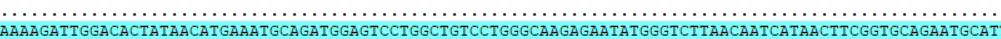

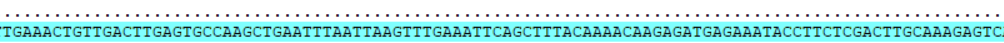

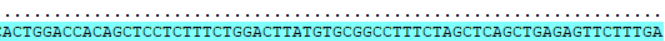


**Supplementary Fig. S14: The alignment of target gene (upper), Unigene11344 and *C.songorica gene* (under), Cs2Chr10028121.**


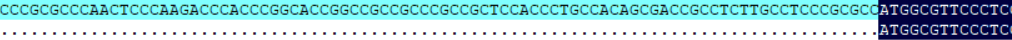

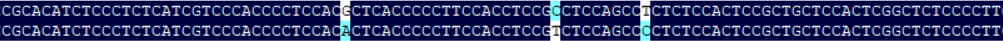

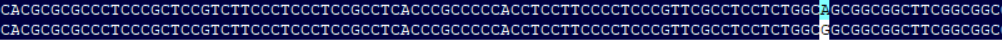


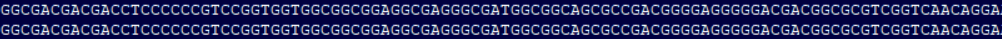

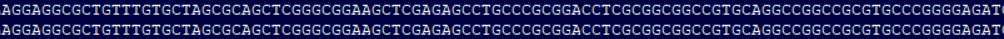

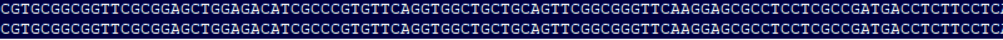

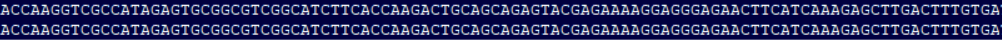

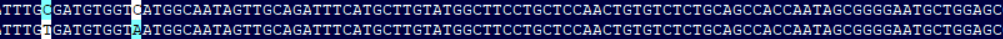

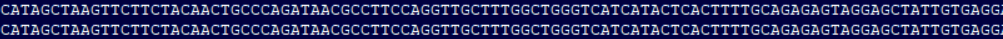

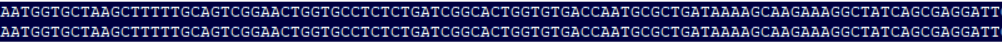

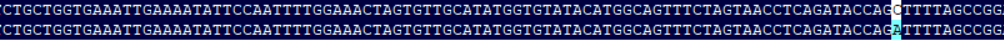

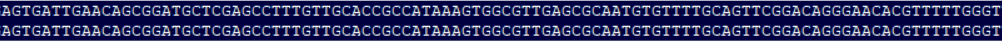

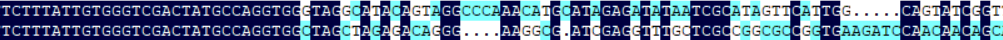

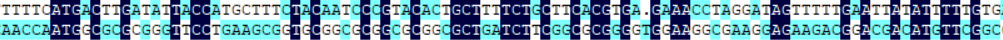

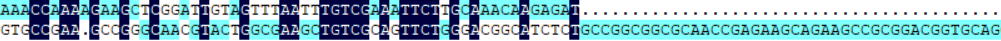

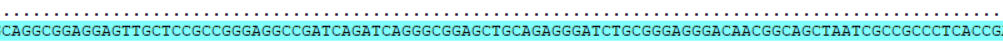

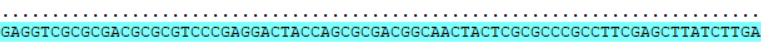


**Supplementary Fig. S15: The alignment of target gene (upper), Unigene13142 and *C.songorica gene* (under), Cs5Chr10051664.**


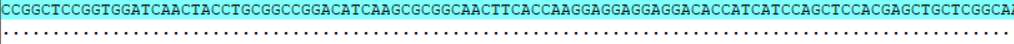

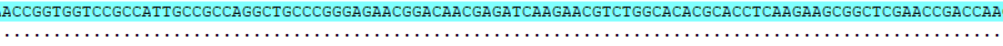

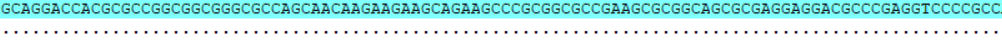

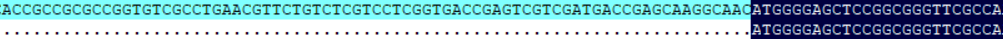

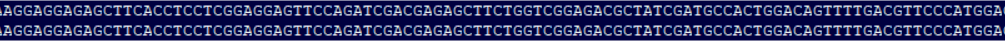

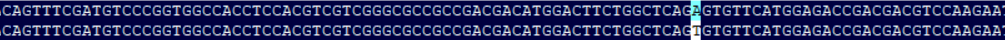

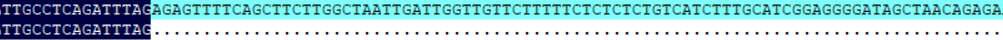

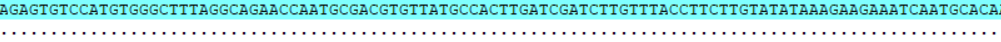

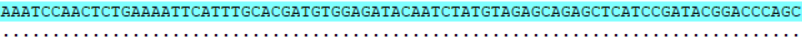


**Supplementary Fig. S16: The alignment of target gene (upper), CL1107.Contig1 and *C.songorica gene* (under), Cs1Chr10054356.1.**


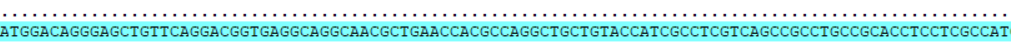

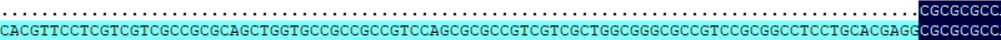

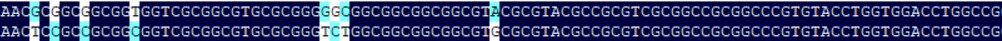

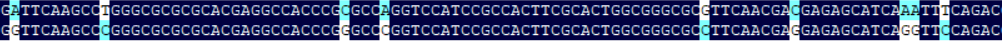

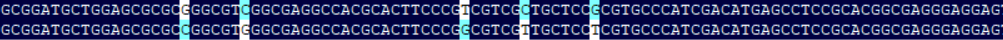

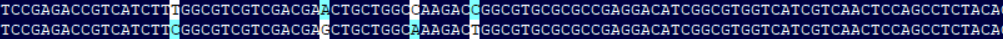

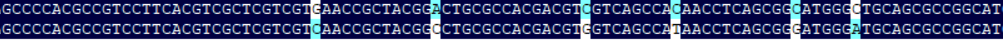


**Supplementary Fig. S17: The alignment of target gene (upper), CL2033.Contig1 and *C.songorica gene* (under), Cs9Chr10036012.**

**Supplementary Fig. S18: The alignment of target gene (upper), CL8003.Contig1 and *C.songorica gene* (under), Cs9Chr10052551.**

**Supplementary Fig. S19: The alignment of** **target gene (upper), CL12849.Contig1 and *C.songorica gene* (under), Cs2Chr10003462.**

**Supplementary Fig. S20: The alignment of** **target gene (upper), Unigene10926_All and *C.songorica gene* (under), Cs88contig10004313.**

**Table S1 Classification of small RNAs reads in four different positions.**

|  | CH | |  | CL_U | |  | CL_M | |  | CL_B | |
| --- | --- | --- | --- | --- | --- | --- | --- | --- | --- | --- | --- |
|  | Total reads (%) | Unique reads (%) |  | Total reads (%) | Unique reads (%) |  | Total reads (%) | Unique reads (%) |  | Total reads (%) | Unique reads (%) |
| miRNA | 28059 (0.25%) | 4567 (0.37%) |  | 43585 (0.4%) | 5845 (0.34%) |  | 72209 (0.67%) | 6783 (0.34%) |  | 25315 (0.24%) | 5927 (0.41%) |
| rRNA | 7429677 (65.04%) | 225941 (18.48%) |  | 4830991 (44.78%) | 177666 (10.35%) |  | 4045291 (37.72%) | 171548 (8.54%) |  | 7041128 (65.45%) | 252461 (17.51%) |
| rRNA | 655764 (5.74%) | 6007 (0.49%) |  | 1428805 (13.24%) | 4666 (0.27%) |  | 1554883 (14.5%) | 4901 (0.24%) |  | 704580 (6.55%) | 5137 (0.36%) |
| snRNA | 80239 (0.7%) | 3102 (0.25%) |  | 7741 (0.07%) | 1157 (0.07%) |  | 7068 (0.07%) | 1221 (0.06%) |  | 11759 (0.11%) | 1527 (0.11%) |
| snoRNA | 29071 (0.25%) | 2356 (0.19%) |  | 22037 0.24% | 1751 (0.1%) |  | 17226 (0.16%) | 1700 (0.08%) |  | 26352 (0.24%) | 2130 (0.15%) |
| tRNA | 780978 (2.37%) | 29014 (6.84%) |  | 329211 (3.05%) | 16464 (0.96%) |  | 334085 (3.12%) | 18243 (0.91%) |  | 366944 (3.41%) | 19352 (1.34%) |
| Unannotation | 2419445 (77.84%) | 951699 (21.18%) |  | 4126951 (38.25%) | 1508724 (87.91%) |  | 4693072 (43.76%) | 1805061 (89.83%) |  | 2581695 (24%) | 1155643 (80.13%) |
| Total | 11423233 | 1222686 |  | 10789321 | 1716273 |  | 10723834 | 2009457 |  | 10757773 | 1442177 |

**Table S2 Summary of assembly quality for *C. songorica*.**

| Sample | Total Reads | Total Nucleotides(nt) | Q20 percentage | N percentange | GC percentage |
| --- | --- | --- | --- | --- | --- |
| CSCL | 77,812,146 | 7,003,093,140 | 97.28% | 0.00% | 51.74% |
| CSCH | 83,092,310 | 7,478,307,900 | 97.62% | 0.00% | 52.06% |

**Table S3 Expression level of know miRNAs.**

**Table S4 Expression level of know miRNAs.**

**Table S5 Summary of miRNA and the target genes in each library.**

|  | miRNA number | |  | Target gene number | | |
| --- | --- | --- | --- | --- | --- | --- |
|  | Known | Novel |  | | Known | Novel |
| CH | 78 | 27 |  | 1657 | | 494 |
| CL_B | 73 | 22 |  | 1776 | | 372 |
| CL_M | 99 | 43 |  | 1868 | | 727 |
| CL_U | 90 | 54 |  | 1407 | | 763 |

**Table S6 GO analysis showing that targets of differentially expressed miRNAs from *Cleistogenes songorica* flower were involved in biological processes.**

| Target genes name | CSCH_FPKM | CSCL_FPKM | GO-BiologicalProcess |
| --- | --- | --- | --- |
| CL6263.Contig1 | 4.9157 | 14.4348 | GO:0009911//positive regulation of flower development |
| CL6263.Contig2 | 2.3516 | 23.1507 | GO:0009911//positive regulation of flower development |
| Unigene18183 | 19.0342 | 41.8686 | GO:0009909//regulation of flower development |
| Unigene8718 | 38.6227 | 9.2483 | GO:0009908//flower development |
| CL12608.Contig1 | 0.3481 | 7.8452 | GO:0009908//flower development |
| Unigene13142 | 4.2441 | 13.6515 | GO:0010229//inflorescence development; GO:0009911//positive regulation of flower development |
| Unigene429 | 127.8411 | 24.1249 | GO:0009856//pollination; GO:0009909//regulation of flower development; GO:0010228//vegetative to reproductive phase transition of meristem; GO:0048653//anther development; GO:0009793//embryo development ending in seed dormancy |
|  |  |  |  |
| Unigene6664 | 9.8602 | 20.5798 | GO:0009909//regulation of flower development |
| CL12443.Contig1 | 4.2441 | 13.6515 | GO:0010584//pollen exine formation; GO:0009911//positive regulation of flower development; GO:0048657//tapetal cell differentiation |
| Unigene11344 | 2.1211 | 15.4812 | GO:0048578//positive regulation of long-day photoperiodism, flowering; GO:0048481//ovule development |
| Unigene13523 | 6.7605 | 13.819 | GO:0048833//specification of floral organ number; GO:0009887//organ morphogenesis; GO:0010080//regulation of floral meristem growth |
| Unigene22705 | 0.225 | 4.1242 | GO:0010077//maintenance of inflorescence meristem identity; GO:0010582//floral meristem determinacy |
| CL10738.Contig2 | 2.936 | 23.8433 | GO:0010582//floral meristem determinacy; GO:0010254//nectary development; GO:0048439//flower morphogenesis; GO:0010227//floral organ abscission |
|  |  |  |  |
| Unigene35106 | 3.1307 | 0.7233 | GO:0048439//flower morphogenesis; GO:0010227//floral organ abscission |
| CL1873.Contig1 | 16.2881 | 3.6318 | GO:0048657//tapetal cell differentiation |
| CL1873.Contig2 | 12.2946 | 3.2361 | GO:0048657//tapetal cell differentiation |
| Unigene21112 | 29.9774 | 9.7702 | GO:0010109//regulation of photosynthesis |
| CL334.Contig6 | 2.3797 | 5.9449 | GO:0006355//regulation of transcription, DNA-dependent |
| Unigene11026 | 14.869 | 6.309 | GO:0006351//transcription, DNA-dependent |

**Table S7 The identified miR156 and 159 family members and their targets with differential expression in *C. songorica***

| miRNA name |  | log2(CL_ /CH) | p-value | Gene-ID | log2(CL/CH) | p-value | GO-annotation |
| --- | --- | --- | --- | --- | --- | --- | --- |
| miR156k | CHvsCL_U | -0.37 | 3.29E-11 | Unigene10926 | -1.52 | 6.94E-19 | - |
|  | CHvsCL_M | -12 | 4.32E-185 |  |  |  |  |
|  | CHvsCL_B | -11.52 | 4.03E-164 |  |  |  |  |
| miR156a-5p | CHvsCL_B | 11.92 | 0 | CL8424.Contig1 | 1.08 | 3.42E-251 | - |
|  | CHvsCL_M | 12.04 | 4.53E-295 | CL1102.Contig1 | -1.53 | 4.96E-05 | - |
|  |  |  |  | CL1954.Contig2 | 2 | 5.83E-54 | response to high light intensity |
| miR156k |  |  |  | Unigene21580 | -1.38 | 0.00012 | protein kinase activity |
| miR156a-5p |  |  |  | Unigene12423 | -1.19 | 0.00041 | protein serine/threonine/tyrosine kinase activity |
|  |  |  |  | CL5893.Contig2 | 1.33 | 1.32E-21 | binding |
|  |  |  |  | CL203.Contig1 | 1.53 | 2.92E-74 | nucleic acid binding |
|  |  |  |  | CL1753.Contig1 | 1.69 | 0.00033 | DNA binding |
|  |  |  |  | Unigene16969 | 1.86 | 1.59E-19 | DNA binding |
|  |  |  |  | CL12497.Contig3 | 1.93 | 7.36E-24 | regulation of vegetative phase change; DNA binding |
|  |  |  |  | CL8003.Contig1 | 2.37 | 1.70E-135 | DNA binding |
|  |  |  |  | CL5893.Contig1 | 2.79 | 7.16E-150 | DNA binding |
|  |  |  |  | CL12497.Contig1 | 2.91 | 1.12E-51 | regulation of vegetative phase change; DNA binding |
|  |  |  |  | CL1753.Contig2 | 13.14 | 1.12E-42 | DNA binding, methyltransferase activity |
| miR156d-3p | CHvsCL_U | 4.93 | 1.71E-06 | Unigene2897 | -3.95 | 2.08E-131 | threonine-type endopeptidase activity |
|  | CHvsCL_B | 4.34 | 0.0004927 |  |  |  |  |
| miR156h-3p | CHvsCL_M | 4.45 | 5.62E-05 | Unigene17355 | 2 | 1.80E-88 | - |
| miR159b-3p | CHvsCL_U | -0.24 | 8.59E-41 | CL12849.Contig1 | 1.19 | 7.50E-153 | amino acid transport |
|  | CHvsCL_M | -3.38 | 0 |  |  |  |  |
|  | CHvsCL_B | -0.17 | 1.64E-18 |  |  |  |  |
| miR159a.1 | CHvsCL_U | -0.27 | 2.40E-62 | CL1107.Contig1 | -3.11 | 3.40E-12 | - |
|  | CHvsCL_M | 0.21 | 1.21E-47 | CL1107.Contig2 | -1.04 | 3.65E-16 | chromatin binding;DNA binding |
|  | CHvsCL_B | -0.22 | 7.34E-35 | CL1107.Contig3 | -3.08 | 3.56E-39 | - |
|  |  |  |  | CL11879.Contig4 | 2.45 | 4.06E-10 | developmental process involved in reproduction |
|  |  |  |  | CL12461.Contig2 | -3.78 | 5.56E-22 | binding |
|  |  |  |  | CL2033.Contig1 | -1.74 | 1.93E-144 | - |
|  |  |  |  | CL2284.Contig1 | -2.04 | 5.78E-87 | - |
|  |  |  |  | CL3459.Contig6 | -1.75 | 6.28E-218 | amino-terminal vacuolar sorting propeptide binding |
|  |  |  |  | CL3996.Contig2 | 1.35 | 1.31E-268 | anther development |
|  |  |  |  | CL424.Contig1 | -4.45 | 3.52E-246 | binding |
|  |  |  |  | Unigene22419 | 1.53 | 2.97E-05 | - |

**Table S8 Summary of miRNA-target pairs in GO pathway analysis.**

**Table S8a miRNA-target pairs and their corresponding number.**

| miRNA-target pair number | miRNA | Target gene |
| --- | --- | --- |
| 1 | miR529-5p | CL13498.Contig3 |
| 2 | miR5054 | CL1873.Contig2 |
|  |  | CL1873.Contig1 |
| 3 | miR5072 | CL1873.Contig2 |
|  |  | CL1873.Contig1 |
| 4 | miR169l-5p | Unigene29333 |
| 5 | miR6196 | Unigene14451 |
| 6 | miR408d | Unigene14451 |
| 7 | miR2592bm-3p | Unigene594 |
| 8 | miR2275d-5p | CL1472.Contig3 |
| 9 | miR5783 | Unigene20701 |

**Table S8b miRNA-target pairs information of GO annotation**

| Term ID | Gene Onthology category | the number of miRNA-target gene | | |
| --- | --- | --- | --- | --- |
|  |  | CH | CL_B | CL_U |
| GO:0032502 | developmental process | 1,2,3,4,5 | 1,2,3,5,6,7,8 | 1,2,3,5,6,9 |
| GO:0044767 | single−organism development | 1,2,3,5 | 1,2,3,5,6,7,8 | 1,2,3 |
| GO:0048856 | anatomical structure a | 2,3 | 2,3,7,8 | 2,3 |
| GO:0007275 | multicellular organism | 1,2,3,5 | 1,2,3,5,6,8 | 1,2,3 |
| GO:0048731 | system development | 2,3 | 2,3,7,8 | 2,3 |
| GO:0009653 | anatomical structure b | 2,3 | 2,3 | 2,3 |
| GO:0009791 | post.embryonic development | 1,2,3 | 1,2,3,7,8 | 1,2,3 |
| GO:0003006 | developmental process | 2,3,4 | 2,3,7 | 2,3,9 |
| GO:0048367 | shoot system development | 2,3 | 2,3,7,8 | 2,3 |
| GO:0061458 | reproductive system | 2,3 | 2,3,7 | 2,3 |
| GO:0048513 | organ development | 2,3 | 2,3,8 | 2,3 |
| GO:0009886 | post.embryonic morphogenesis | 2,3 | 2,3 | 2,3 |
| GO:0048608 | reproductive structure | 2,3 | 2,3,7 | 2,3 |
| GO:0048569 | post−embryonic organism a | 2,3 | 2,3,8 | 2,3 |
| GO:0009887 | organ morphogenesis | 2,3 | 2,3 | 2,3 |
| GO:0048563 | post−embryonic organism b | 2,3 | 2,3 | 2,3 |
| GO:0009908 | flower development | 2,3 | 2,3,7 | 2,3 |
| GO:0048437 | floral organ development | 2,3 | 2,3 | 2,3 |
| GO:0048444 | floral organ morphogenesis | 2,3 | 2,3 | 2,3 |

**Table S9 potential precursors and primers of 15 miRNAs used for qRT-PCR analysis.**

| miRNA name | Mature sequence | Primer sequence (5' to 3') | Primer length | Primer GC% | Primer TM °C |
| --- | --- | --- | --- | --- | --- |
| novel_mir_16 | CUUCUGAAAAGUUGUGGCACG | CTTCTGAAAAGTTGTGGCACG | 21 | 47.6 | 54.6 |
| novel_mir_10 | AGCGGGCGAGGAAGGCGGCGCC | TAGCGGGCGAGGAAGGC | 17 | 70.6 | 61.7 |
| novel_mir_34 | CAAGUUAUGCAGUUGCUGCCU | CAAGTTATGCAGTTGCTGCCT | 21 | 47.6 | 55.9 |
| novel_mir_13 | GUUGUUCUCGUCGAAGUCAGC | AGTTGTTCTCGTCGAAGTCAG | 21 | 47.6 | 54.4 |
| miR9484 | UAGGCAAGGGAAGUCGGC | ATAGGCAAGGGAAGTCGGC | 19 | 57.9 | 57.8 |
| miR7984a | UCCGACUUUGUGAAAUGACUU | CGTCCGACTTTGTGAAATGACTT | 23 | 43.5 | 55.3 |
| miR528-5p | GGAAGGGGCAUGCAGAGGAG | GGAAGGGGCATGCAGAGG | 18 | 66.7 | 59.8 |
| miR5077 | UUCACGUCGGGUUCACCA | CTTCACGTCGGGTTCACCA | 19 | 57.9 | 57.9 |
| miR5021 | UGAGAGAAGAGGAAGAACAC | GTGAGAGAAGAGGAAGAACAC | 21 | 47.6 | 52.3 |
| miR408d | UGCACUGCCUCUUCCCUGGC | ATGCACTGCCTCTTCCCTG | 19 | 57.9 | 58 |
| miR408-3p | CUGCACUGCCUCUUCCCUGGC | TCTGCACTGCCTCTTCCCT | 19 | 57.9 | 59 |
| miR319b-3p | UUGGACUGAAGGGUGCUCCCU | TTGGACTGAAGGGTGCTCC | 19 | 57.9 | 57.6 |
| miR319b | UUGGACUGAAGGGUGCUCCC | TTGGACTGAAGGGTGCTCC | 19 | 57.9 | 57.6 |
| miR2907a | GACGCCGGCGGGGGCCUCGG | AGACGCCGGCGGGGG | 15 | 86.7 | 67.1 |
| miR159a.1 | CUUGGAUUGAAGGGAGCUCUG | CTTGGATTGAAGGGAGCTCTG | 21 | 47.6 | 54.7 |
| U6 |  | Forward: GGGGACATCCGATAAAATT Reverse: TGTGCGTGTCATCCTTGC |  |  |  |

**Table S10 Primers information of the 17 target genes used for qRT-PCR analysis.**

| target genes name | Sequence (5' to 3') | length | GC% | TM °C |
| --- | --- | --- | --- | --- |
| CL10738.Contig2F | GGAGGTAATGGTGCGTCATG | 20 | 55 | 56.3 |
| CL10738.Contig2R | GTCGCCAAGATCGAGGAGA | 19 | 57.9 | 57 |
| CL12608.Contig1F | CGAAGTCTGTGAGCATGATGTG | 22 | 50 | 56.1 |
| CL12608.Contig1R | TCGCCTCCGCAAGGTTCTA | 19 | 57.9 | 59.1 |
| CL334.Contig6F | CATGGTTGCTTCAGTCGTCTTC | 22 | 50 | 56.3 |
| CL334.Contig6R | GCAGGCGATGATGGATGGAA | 20 | 55 | 58.2 |
| CL6263.Contig1F | ACCTCATCTCGCTGCCCAA | 19 | 57.9 | 59.6 |
| CL6263.Contig1R | AACGCTTCGCGGTTCTTGA | 19 | 52.6 | 57.7 |
| CL6263.Contig2F | CCCAAGGACTTTGCCCACT | 19 | 57.9 | 58.3 |
| CL6263.Contig2R | TCGATGACGCGGAATGCT | 18 | 55.6 | 57.3 |
| Unigene11026F | AGCCGTAGAAGCGATAGTCA | 20 | 50 | 55.4 |
| Unigene11026R | GCCATGCTCACCATCAGAC | 19 | 57.9 | 56.7 |
| Unigene13142F | GTGGTCCAGGTCCTCTTCTTC | 21 | 57.1 | 57.6 |
| Unigene13142R | GTCCGCCTTCCAGTCATCAT | 20 | 55 | 57.5 |
| Unigene13523F | GATGATGTTGTCCTCCTTGA | 20 | 45 | 51.5 |
| Unigene13523R | GTGATCGTGTTCGCTGTA | 18 | 50 | 52 |
| Unigene18183F | TGGACTGCCTTGCGTTGA | 18 | 55.6 | 57.5 |
| Unigene18183R | AGCGGAACCAGCCATTGA | 18 | 555.6 | 57.4 |
| Unigene35106F | CGATGTCGGTGTGGATTCC | 19 | 57.9 | 56.3 |
| Unigene35106R | GCTGAAGGAGATGCGAGAC | 19 | 57.9 | 56 |
| Unigene429F | TCGTTCCCGGAGCTGTAAG | 19 | 57.9 | 57.2 |
| Unigene429R | GGTCCCAGGGCAAGAATTTC | 20 | 55 | 56.6 |
| Unigene6664F | TGCGTATGTTGCTCAGGTTG | 20 | 50 | 55.7 |
| Unigene6664R | GGTTGTGCTGCGACTATAAGG | 21 | 52.4 | 56.1 |
| Unigene8718F | GCCAGCAGAGGAACTTGAAC | 20 | 55 | 56.7 |
| Unigene8718R | GCCGAGGAACAGTGGTCTT | 19 | 57.9 | 57.8 |
| CL12443.Contig1F | CTCGTCGCTCTGCCAAGAA | 19 | 57.9 | 58 |
| CL12443.Contig1R | CGTTGATGAGGTTGAGACTGAG | 22 | 50 | 55.4 |
| Unigene11344F | CGCCTGTACGACTTCTCCA | 19 | 57.9 | 57.2 |
| Unigene11344R | CAATCTTGCTGCCTCTGCTT | 20 | 50 | 55.8 |
| Unigene21112F | AGGACAGGAAGGTGGTGGA | 19 | 57.9 | 58.5 |
| Unigene21112R | CGTCAGGAATGGCGGAAGA | 19 | 57.9 | 57.9 |
| Unigene22705F | GCAAGAAGCAGGCGAAGAAG | 20 | 55 | 57.2 |
| Unigene22705R | TCGATCTCGTCCTCCAGCA | 19 | 57.9 | 58.3 |
| CsGAPDHF | GTCAGCCAAGGACTGGAGAG | 20 | 60 | 58.1 |
| CsGAPDHFR | ACACATCGACTGTTGGGACA | 20 | 50 | 56.3 |

**Table S11 miRNA and mdm-miRNA relative expression.**

|  | Log2(CH/CL_) | | |  | Log2(CH/CL_) | | |
| --- | --- | --- | --- | --- | --- | --- | --- |
| miRNA (No reference genome) | CHvsCL_U | CHvsCL_M | CHvsCL_B | miRNA (Reference genome) | CHvsCL_U | CHvsCL_M | CHvsCL_B |
| >miR408d | 5.38 | 6.32 | 3.99 | >mdm-miR408a | -1.58 | -1.00 | -1.58 |
| TGCACTGCCTCTTCCCTGGC |  |  |  | ATGCACTGCCTCTTCCCTGGC |  |  |  |
| >miR408-3p | 5.22 | 6.20 |  |  |  |  |  |
| CTGCACTGCCTCTTCCCTGGC |  |  |  |  |  |  |  |
| >miR156a-5p |  | 12.04 | 11.92 | >mdm-miR156a | -0.51 | -0.78 | -0.51 |
| TGACAGAAGAGAGTGAGCAC |  |  |  | TGACAGAAGAGAGTGAGCAC |  |  |  |
| >miR156k | -0.37 | -12.00 | -11.52 | >mdm-miR156t | -1.00 | -1.32 | -0.58 |
| TTGACAGAAGAGAGTGAGCAC |  |  |  | TTGACAGAAGAGAGAGAGCAC |  |  |  |
| >miR159a.1 | -0.27 | 0.21 | -0.22 | >mdm-miR159a | -0.49 | -0.49 | -0.49 |
| CTTGGATTGAAGGGAGCTCTG |  |  |  | CTTGGATTGAAGGGAGCTCC |  |  |  |
| >miR159b-3p | -0.24 | -3.38 | -0.17 | >mdm-miR159b | -0.49 | -0.49 | -0.49 |
| CTTGGATTGAAGGGAGCTCTGT |  |  |  | CTTGGATTGAAGGGAGCTCC |  |  |  |

#### Table 12 miRNA target gene [corresponding to](javascript:;) *C.songorica* gene (reference genome).

|  | Target gene | C. songorica gene |
| --- | --- | --- |
| miR159a.1 | CL1107.Contig1 | Cs1Chr10054356 |
|  | CL1107.Contig2 | Cs10Chr10007141 |
|  | CL12461.Contig2 | Cs1305contig10022700 |
|  | CL2033.Contig1 | Cs9Chr10036012 |
|  | CL2284.Contig1 | Cs9Chr10012436 |
|  | CL3996.Contig2 | Cs11Chr10033577 |
|  | Unigene22419 | Cs6Chr10049639 |
| miR156a-5p | CL8424.Contig1 | Cs2Chr10032109 |
|  | CL1102.Contig1 | Cs3Chr10048690 |
|  | CL1954.Contig2 | Cs2Chr10043435 |
| miR156a-5p | Unigene21580 | Cs1Chr10054317 |
| miR156k | Unigene12423 | Cs9Chr10005158 |
|  | CL5893.Contig2 | Cs8Chr10047255 |
|  | CL203.Contig1 | Cs9Chr10042186 |
|  | CL1753.Contig1 | Cs9Chr10043934 |
|  | Unigene16969 | Cs5Chr10031039 |
|  | CL12497.Contig1 | Cs1Chr10052329 |
|  | CL8003.Contig1 | Cs9Chr10052551 |
|  | CL5893.Contig1 | Cs8Chr10047255 |
| miR156k | Unigene10926 | Cs88contig10004313 |
| miR156d-3p | CL12849.Contig1 | Cs2Chr10003462 |
| miR5054 | CL1873.Contig1 | Cs3Chr10009316 |
| miR5072 | CL1873.Contig2 | Cs3Chr10009316 |
| miR5077 | CL6263.Contig1 | Cs1Chr10017715 |
| miR5077 | CL6263.Contig2 | Cs1Chr10017715 |
| miR2907a | CL12608.Contig1 | Cs1Chr10030343 |
| miR408d-3p | Unigene429 | Cs323contig10000755 |
| miR408d |  |  |
| miR528-5P | Unigene6664 | Cs3Chr10014223 |
| miR319b | Unigene8718 | Cs8Chr10045326 |
| miR319b-3p | Unigene11026 | Cs3Chr10033236 |
| miR2907a | Unigene35106 | Cs6Chr10051849 |
| miR5021 | Unigene13142 | Cs5Chr10051664 |
| miR9484 | Unigene13523 | Cs2Chr10044831 |
| novel_mir_10 | Unigene22705 | Cs1Chr10032885 |
| novel_mir_16 | Unigene21112 | Cs5Chr10031073 |
| novel_mir_34 | Unigene11344 | Cs2Chr10028121 |
